# Synthesis and appraisal of dalbergin-loaded PLGA nanoparticles modified with galactose against hepatocellular carcinoma: *In-vitro*, pharmacokinetic, and *in-silico* studies

**DOI:** 10.3389/fphar.2022.1021867

**Published:** 2022-10-28

**Authors:** Anurag Kumar Gautam, Pranesh Kumar, Biswanath Maity, Ganesh Routholla, Balaram Ghosh, Kumarappan Chidambaram, M. Yasmin Begum, Adel Al Fatease, P.S. Rajinikanth, Sanjay Singh, Sudipta Saha, Vijayakumar M. R.

**Affiliations:** ^1^ Department of Pharmaceutical Sciences, Babasaheb Bhimrao Ambedkar University, Lucknow, Uttar Pradesh, India; ^2^ Department of Pharmacology, Aryakul College of Pharmacy & Research, Lucknow, Uttar Pradesh, India; ^3^ Centre of Biomedical Research, SGPGIMS Campus, Lucknow, Uttar Pradesh, India; ^4^ Department of Pharmacy, BITS-Pilani Hyderabad Campus Hyderabad, Hyderabad, India; ^5^ Department of Pharmacology and Toxicology, School of Pharmacy, King Khalid University, Abha, Saudi Arabia; ^6^ Department of Pharmaceutics, King Khalid University, Abha, Saudi Arabia

**Keywords:** dalbergin, HCC, galactose decorated nanoparticles, targeted nanoparticles, liver cancer targeting

## Abstract

Hepatocellular carcinoma (HCC) is a common malignancy which affects a substantial number of individuals all over the globe. It is the third primary cause of death among persons with neoplasm and has the fifth largest mortality rate among men and the seventh highest mortality rate among women. Dalbergin (DL) is described to be effective in breast cancer *via* changing mRNA levels of apoptosis-related proteins. DL belongs to neoflavonoids, a drug category with low solubility and poor bioavailability. We created a synthetic version of this naturally occurring chemical, DL, and then analyzed it using ^1^H-NMR, ^13^C-NMR, and LC-MS. We also made PLGA nanoparticles and then coated them with galactose. The design of experiment software was used to optimize DL-loaded galactose-modified PLGA nanoparticles. The optimized DL-nanoformulations (DLF) and DL-modified nanoformulations (DLMF) were analyzed for particle size, polydispersity index, shape, and potential interactions. *In-vitro* experiments on liver cancer cell lines (HepG2) are used to validate the anti-proliferative efficacy of the modified DLMF. The *in-vitro* research on HepG2 cell lines also demonstrated cellular accumulation of DLF and DLMF by FITC level. The *in-vitro* result suggested that DLMF has high therapeutic effectiveness against HCC. *In-vivo* pharmacokinetics and bio-distribution experiments revealed that DLMF excelled pristine DL in terms of pharmacokinetic performance and targeted delivery, which is related to galactose’s targeting activity on the asialoglycoprotein receptor (ASGPR) in hepatic cells. Additionally, we performed an *in-silico* study of DL on caspase 3 and 9 proteins, and the results were found to be −6.7 kcal/mol and −6.6 kcal/mol, respectively. Our *in-silico* analysis revealed that the DL had strong apoptotic properties against HCC.

## Graphical Abstract

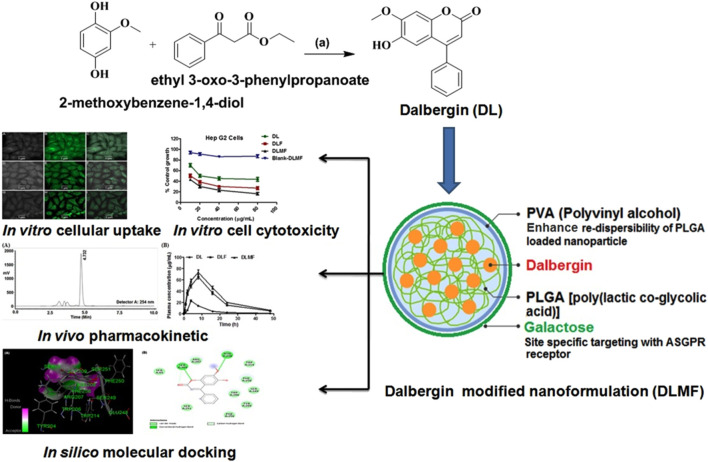

## 1 Introduction

Hepatocellular carcinoma, often known as HCC, is the kind of malignancy with high fatality rate of 5.3 million all over the globe. HCC is the most common type of primary liver cancer, often occurring in people with chronic liver diseases, such as cirrhosis caused by hepatitis B and hepatitis C. HCC is the third most lethal kind of cancer across the globe (D’souza, A.A. and Devarajan, P.V., 2015). In India, the overall number of HCC cases in men is ranging from 0.7 to 7.5 per 1 lakh person per year and it is about 0.2–2.2 in women. Unfortunately, HCC is typically diagnosed late in its course, with a median survival following diagnosis of approximately 6–20 months (Acharya, S.K., 2014). In the United States, 2 years survival is less than 50%, and 5-year survival is only 10%. Sorafenib is the only medication approved for the treatment of HCC, although it has low effectiveness and is susceptible to resistance (Chen, Z., et al.,2020; Llovet, J.M., et al., 2008). Chemotherapeutics are unsuccessful in treating HCC because of their low selectivity for malignant tumor cells. The therapeutic options for HCC have a low survival rate, which may have life-threatening effects because of the failure of tumor selectivity, dose-dependent toxic effects, and the development of resistance to the treatment (Nair, A.B., et al.,2019; Nisha et al., 2020a). For approximately 10 years, the only drug available for advanced hepatocellular carcinoma (HCC) was sorafenib (Nexavar, Bayer), given as first-line treatment (Rimassa, L., 2018). Recently, regorafenib (Stivarga, Bayer) was approved for second-line treatment. In addition, nivolumab (Opdivo, Bristol-Myers Squibb) recently received accelerated approval in the United States for HCC patients who previously used sorafenib and will likely be approved elsewhere based on the clinical outcome (Rimassa, L., 2018). Lenvatinib (Lenvima, Eisai) was approved recently in 80 countries to treat unresectable HCC (https://www.eisai.com/news/2022/news202259.html). Cabozantinib (Cabometyx, Exelixis) will also likely to be approved for second- and third-line treatment soon; if so, this drug would be the first for third-line HCC treatment (Al-Salama, Z.T., et al., 2019). Ramucirumab (Cyramza, Lilly) is also about to be approved. It would be the first HCC drug for a biomarker-selected patient population (second-line treatment of patients with high baseline a-fetoprotein [AFP] values [≥400 ng/ml]) (Bteich, F. and Di Bisceglie, A.M., 2019). Thus, the landscape of HCC treatment is becoming more complex, and it will be important to refine the treatment algorithm for HCC patients. There are several molecular compounds derived from herbs that have been proven to be effective against HCC. Modern research confirms that many compounds are active at some molecular targets, which are being sought to discover potential newer generation targeted biological response modifier drugs. These herbal compounds have been shown to engage various molecular targets related to HCC carcinogenesis and chemoprevention as reported in the research findings and clinical observations. These molecular compounds represent an enormous and almost untapped resource for HCC treatment (Li, Y. and Martin, R.C., 2011). Neoflavonoids derived from natural sources are categorized as polyphenolic compounds, in contrast to flavonoids containing a 2-phenylchromen-4-one backbone. The molecular structure of neoflavonoids contains a 4-phenylchromen framework, and there is no hydroxyl group present in the second position of the molecule. Neoflavonoids have been shown to be effective in a variety of settings, including those pertaining to the prevention of cancer, the reduction of inflammation, and the enhancement of antioxidant properties (Mahdizade, V.F., et al., 2019). For developing semi-synthetic derivatives with better pharmacodynamics and pharmacokinetics of the dalbergin that belongs to the neoflavonoids category although this compound has lower solubility and bioavailability. Dalbergin (DL) is a natural product found in *Dalbergia cochinchinensis*, *Pterocarpus santalinus*, and other organisms. Several publications show the broad spectrum of DL, such as antimicrobial, antiplasmodial, osteogenic, antifungal, anti-inflammatory, antidiabetic, anticancer, antimycobacterial, cardiovascular, cytotoxicity, *etc.* (Kumar, P., et al., 2017). Poly (lactic-co-glycolic acid) (PLGA) is one of the most successfully developed biodegradable polymers. Among the different polymers developed to formulate polymeric nanoparticles, PLGA has attracted considerable attention due to its attractive properties: biodegradability and biocompatibility (Amirsaadat, S., et al., 2021), FDA and European Medicine Agency approval in drug delivery systems for parenteral administration (Makadia, H.K. and Siegel, S.J., 2011), well-described formulations and methods of production adapted to various types of drugs, e.g., hydrophilic or hydrophobic small molecules or macromolecules, protection of the drug from degradation, possibility of sustained release, possibility to modify surface properties to provide better interaction with biological materials and a possibility to target nanoparticles to specific organs or cells (Makadia, H.K. and Siegel, S.J., 2011; Danhier, F., et al., 2012). Galactose (GL) is a monosaccharide employed to modify the surface of nanoparticles in this study. GL binds with the asialoglycoprotein receptors (ASGPR) and interacts with the destination (Li, Y., et al., 2008; Bakrania, A., et al., 2021). GL-decorated PLGA nanoparticle mediated targeting was shown to be effective in exhibiting targeted drug delivery to the tumors, avoidance of complete removal through the reticuloendothelial system (RES) (Adlravan, E., et al., 2021), and enhanced permeability and retention (EPR) impact at the HCC region. Due to the high levels of ASGPR expression in the hepatic cancer tissue, these receptors offer an improved choice for delivery of drugs in the treatment of HCC (Makadia, H.K. and Siegel, S.J., 2011; Huang, K.W., et al., 2018). We developed (PLGA) poly (lactic-co-glycolic acid) nanoparticles with decorations to address the restricted solubility and effectiveness of drug administration. Several research works showed that PLGA is a biodegradable polymer (Samadzadeh, S., et al., 2021) that minimizes drug toxicity and promotes cellular absorption of drugs by interacting with site-specific interactions that boost target specificity and enhance efficiency (Makadia, H.K. and Siegel, S.J., 2011; Gupta, S., et al., 2013).

Therefore, in this study, for the first time, we attempted to develop new PLGA nanoparticles of DL. We applied Box-Behnken design (BBD) statistical-based formulation design to optimize DL-loaded PLGA nanoparticles. Also, the surface modification of DLF with galactose was attempted to prepare galactose-decorated PLGA nanoparticles for the DL release on the hepatocytes cell. Further, DL, DLF, and DLMF were assessed for anti-HCC capacity *via in-vitro* (HepG2 cell lines) study. Moreover, the *in-vivo* biodistribution of DL, DLF, and DLMF were explored through High performance liquid chromatography (HPLC) analysis to evaluate their targeting potential in detail (Gupta, S., et al., 2013; Amjadi, I., et al., 2013). Lastly *in-silico* analysis carried out for first time to analyze the apoptotic properties of DL against HCC through caspases.

## 2 Materials and procedures

### 2.1 Materials

PLGA [poly lactic-co-glycolic acid (50:50)], Poly Vinyl Alcohol (PVA), Ethylenediamine (EDA), and 1-ethyl-3-(-3-dimethylaminopropyl) carbodiimide (EDC), Fluorescein isothiocyanate (FITC), were supplied by Thermo Fisher Scientific India Pvt. Ltd. Purchases of DMEM and FBS were made from Sigma-Aldrich of Burlington, MA, United States. Researchers opted to utilize double-distilled water (ddH_2_O) (sourced from Borosil, India) throughout the experiment. All of the compounds utilized were of the analytical class.

### 2.2 Procedures

#### 2.2.1 Synthesis and identification of DL or (6-hydroxy-7-methoxy-4-phenyl-2H-cromen-2-one)

At 0°C, 2-methoxybenzene-1,4-diol (500 mg, 3.57 mmol) was mixed with 15 ml sulphuric acid (75% in H_2_O), and ethyl 3-oxo-3-phenylpropanoate (0.617 ml, 3.57 mmol) was poured dropwise to the phenolic compound solution. The reaction mixture was agitated continuously for half an hour, at room temperature. TLC was used to observe the formation of the compound. After completion of the reaction, the mixture was mixed into ice-cold water and extracted using ethyl acetate. The recovered organic layer was dried using sodium sulphate, and the rotavap was used to evaporate the remaining solvent. The crude product was recrystallized from ethanol (42% yield). ^1^H NMR (400 MHz, DMSO-d_6_) δ: 9.42 (s, 1H), 7.58 (m, 5H), 7.12 (s, 1H), 6.82 (s, 1H), 6.19 (s, 1H), 3.89 (s, 3H). ^13^C NMR (101 MHz, DMSO-d_6_) δ: 160.86, 155.59, 152.41, 149.01, 144.05, 135.82, 130.00, 129.30, 128.80, 111.60, 110.82, 100.89, 56.65. Calculated C_16_H_12_O_4_ [M]: 268.26; found mass, (ESI) m/z: [M-H]^-^ 266.87 (Kawase M, et al., 2005) (Supplementary Figures SA, SB, SC).

### 2.3 Experimental design for optimization of DLF

The formulation and process variables were statistically optimized using BBD. Design Expert has been used to design DLF utilizing a three-factor system, each at three levels (Trial version 8.0 of Design-Expert software). On response factors like particle size and entrapment efficiency (EE), the effects of independent factors such as concentration of PLGA (X1), PVA (X2), and sonication time (X3) were studied. To create DL with a particle size range of 100–150 nm and the highest feasible EE score, independent factors required to be investigated on DLF-dependent attributes. Three trials were conducted to assure accurate findings. The experimental design that was ultimately selected brought in a decrease in the overall quantity of trials, a reduction in the amount of time and dedication needed, as well as an increase in the amount of money saved (Nisha et al., 2020a).

Based on the results of several early tests, 15 different DL-loaded nanoformulations (DLF) were developed by altering the three parameters: the amount of PLGA, the amount of PVA, and the amount of time spent sonicating at one of three different intensities: high (+1), intermediate (0), and low (1). The formula was developed based on stated factors, such as having the maximum EE attraction and the lowest particle size attraction. The optimized formulation was chosen based on the preset criterion of highest %EE and least particle size. We assessed the changes in response parameters and chose the optimized formulation with the highest desirability (near 1), subsequently creating the formulation in three copies to guarantee the accuracy of the optimization (Nisha et al., 2020b).

### 2.4 Preparation of galactose-modified PLGA nanoparticles

#### 2.4.1 Synthesis of galactose-PLGA conjugate

PLGA was conjugated to ethylenediamine (EDA). Accordance to a previously published approach with minor adjustments, In summary, 2.5 ml of dH_2_O was added to 50 mg of PLGA and combined on an ice bath at 2000 rev/min. Then 40 μL of EDA was added to the previously described solution (pH-5). The EDC (112 mg) aqueous solution was added to the reaction mixture (pH-5). The reaction mixture was therefore agitated at a low speed (100 rpm) overnight before even being dialyzed for 1 h with ddH_2_O to remove excess EDA.

Amine-terminated PLGA was linked to galactose as described by Gupta, S. *et al.* (2013) with a few minor modifications. Accordingly, 0.1 M sodium acetate buffer (pH-4) containing d-galactose (8 μM) was mixed with amine-terminated PLGA (0.1 mmol). The mixture solution was swirled constantly at room temperature for 48 h to ensure that the reaction was completed successfully. The final solution was dialyzed for 24 h against ddH_2_O to alienate other contaminants and unconjugated galactose before being lyophilized in a dialysis membrane (12 kDa, MWCO, Himedia, Mumbai, India) (Gupta, S., et al., 2013).

#### 2.4.2 Preparation of nanoparticles

Double emulsification was used to synthesize PLGA nanoparticles of DL (DLF) and galactose-modified PLGA nanoparticles (DLMF). The galactose-PLGA conjugate (25 mg) was dissolved in a mixture of dichloromethane and acetone at a ratio of 3:1 v/v. After that, 2.5 mg of DL was mixed in PVA at a concentration of 1% in the solution mixture (0.2 ml). The mixture was then emulsified in an ice bath using sonication at 50 W output for 1 min, resulting in w/o emulsion. In addition, a solution of PVA that was 1% and included 4 ml was mixed drop by drop and sonicated for 2 min over an ice bath. The obtained w/o/w emulsion was further diluted with 10 ml of 0.15% PVA solution, and it was agitated at 1,000 rev/min for 12 h to evaporate the organic solvent. Finally, the resultant nanoparticles were acquired, rinsed with ddH_2_O by centrifugation (30,000 rev/min) for 30 min at 4°C, and lyophilized (Gupta, S., et al., 2013).

### 2.5 Characterization of DLF, and DLMF

#### 2.5.1 Particle size and polydispersity index

Zetasizer Nanoplus (Nanoplus-3) was used to assess DLF and DLMF regarding their particle size and PDI. The functionality of this instrument is supported by the dynamic light scattering technique (Javan, N., et al., 2019; Song, X., et al., 2018). The test nanoformulations were diluted in ddH_2_O before being moved to a cuvette for examination. The particle size and PDI were measured in triplicate, and the results are expressed as mean ± SD (Kanoujia, J., et al., 2021).

### 2.6 Entrapment efficiency and drug loading

Entrapment efficiency is evaluated using ultracentrifugation method. A specific amount of DLF and DLMF were centrifuged at 4°C for 20 min at 15,000 rev/min to isolate the free drug. A UV-Vis Spectrometer (Labtronics-2910, India) set at 254 nm was used to assess the amount of unentrapped drug in the supernatant and to calculate the amount of DL entrapped. We calculated the EE and drug loading using the following formula (Song, X., et al., 2019).

% EE=Initial amount of drug in DLF−free drug/Initial amount of drug in DLF*100 (I)

Drug loading (%)=Amount of drug in lyophilized nanoparticles / Amount of lyophilized nanoparticles×100 (II)

### 2.7 *In vitro* drug release study

At pH values of 1.2 and 7.4, the drug release perspectives of DLF and DLMF were studied in both simulated gastric and simulated intestinal fluids. A flask was placed in an incubator with a water bath continually swirling (100 rev/min) and kept at a temperature of 37°C. The flask was filled with 10 ml of the appropriate medium and weighed amounts of DLF and DLMF that were sufficient for 10 mg of DL. The dissolving solution in the flask was then routinely replaced with new media at regular intervals, after which 2 ml of the sample was collected for DL detection. The samples were analyzed at 254 nm using a UV-Vis Spectrometer (Labtronics-2910, India), which was utilized to quantify the DL release. Triplicate dissolution tests were carried out. The data was analyzed to determine release kinetics (Song, X., et al., 2018; Kanoujia, J., et al., 2021; Song, X., et al., 2019; Kumar, P., et al., 2018).

### 2.8 Drug interaction analysis using FTIR

The FTIR experiments were performed to assess any interaction between DL and polymers. In this experiment, FTIR spectra of DL, DLF, and DLMF were obtained separately. 5 mg of test substances were grounded with 200 mg of potassium bromide (KBr) after pelletizing at 1,000 psi. In the final, the FTIR data were collected and analyzed with the help of a Nicolet™ 6700 from Thermo Fischer Scientific. This particular sensor can detect waves with a wave number range between 4,000 and 500 cm^−1^ (Kumar, P., et al., 2018).

### 2.9 Scanning electron microscopy studies

After the preparation of nanoformulations, we used lyophilizer equipment to lyophilize to harvest nanoparticles. The lyophilized nanoparticles were placed on grids. The JSM-6490LV was used to acquire the SEM pictures (JEOL, Tokyo, Japan) (Kumar, P., et al., 2018).

### 2.10 Stability studies

The ICH, Q1A (R2) criteria were followed for DLMF stability testing. The particle size and EE of the formulation were assessed. To validate the DLMF’s long-term stability, the formulation was kept in clear containers at 4 ± 1°C and 25 ± 2°C conditions, the stability profile of the product was determined. The stored samples at two different temperatures were analyzed on the day of commencing the experiment and on the 30th, 60th, and 90th days (Nisha et al., 2020a; Kumar, P., et al., 2018).

### 2.11 Evaluation of anticancer efficacy

National Cell Repository of NCCS (Pune, India) provided the HepG2 cell line, which was then grown in our cell culture lab for further *in vitro* assays. The liver cancer cell lines were grown in a T-75 flask for 24 h at 37°C, 5% CO_2_, and absolute humidity equal to 100% in the Roswell Park Memorial Institute (RPMI)-1640 media with 10% fetal bovine serum and 2 mM L-glutamine. The cells were collected by centrifugation and incubated at a density of 5×10^3^ cells/well onto 96 well plates to measure the growth. The test samples were distributed in dimethyl sulfoxide at 100 mg/ml concentration, diluted to 1 mg/ml equivalent drug concentration in distilled H_2_O. Each well was treated with 100 μL of the test samples that the organizational culture includes at concentrations of 10, 20, 40, and 80 μg/ml, and they were allowed to incubate for a total of 48 h under the appropriate conditions. 50 μL of cold 30% trichloroacetic acid (TCA) was injected to stop the reaction and incubated for 1 h at a temperature of 4°C. The test plate is washed using fivefold volume of distilled water. In addition, each well was treated with 50 μL of SRB (sulforhodamine B) solution comprising 0.4% (w/v) in 1% acetic acid; it was kept at ambient temperature for 20 min during incubation. Following completion of the staining procedure, the plate was rinsed 5 times with 1% acetic acid to remove color leftover before air drying. After removing the attached dye with 10 mM Trizma base, absorbance was measured at 540 nm using a 690 nm reference. The findings were acquired in triplicate on different plates, and the mean of the three trials was determined. The % cell growth was measured using absorbance data (time zero [Tz], control growth (C), and test growth in the presence of drug at different concentrations [Ti]. The % growth inhibition was calculated using the following formula

$$\frac{Ti-Tz}{C-Tz}\times 100\;for\;concentrations\;for\;which\;Ti\geq Tz$$

$$\frac{Ti-Tz}{C-Tz}\times 100\;for\;concentrations\;for\;which\;Ti\;<Tz$$

It was shown that the quantity of protein formation was reduced by 50% at a medication concentration of 100 = 50. (It is visible due to the SRB staining) in control cells, while the drug was being incubated (Kumar, P., et al., 2018; Liu H, et al., 2016; Ly HT, et al., 2021).

### 2.12 *In vitro* cellular internalization analysis

HepG2 cells were seeded on Lab-Tek chambered cover glasses (Nalge Nunc International, Naperville, IL, United States) and incubated until 70% confluent at 37°C in 95% air and 5% CO_2_. After 30 min of equilibration with HBSS at 37 °C, the buffer was replaced with fluorescein isothiocyanate (FITC)-loaded DL, (FITC)-loaded DLF, and (FITC)-loaded DLMF suspensions (250 mg/ml in HBSS), respectively and the monolayers were incubated for one or 2 h. In the subsequent step, the monolayers were rinsed 3 times with freshly warmed transport buffer to eliminate any nanoparticles that were not internalized into the cells. Following this step, the HepG2 cells were fixed in ethanol at a concentration of 70%, and until evaluation, the samples were mounted in fluorescent mounting media (Dako Denmark A/S, Glostrup, Denmark). Finally, images were captured with the use of a confocal laser scanning microscope (Model: LSM900, Make: Zeiss, Germany) (Nisha et al., 2020b).

### 2.13 Pharmacokinetic studies

#### 2.14.1 Collection of blood

DL, DLF, and DLMF were administered orally to albino Wistar rats at a dose of DL equivalent to 100 mg/kg (Approval No: *IU/IAEC/20/01*), and after the first treatment, the blood samples were collected through retro-orbital plexus of the animals at 0.083, 0.25, 0.5, 1, 2, 4, 8, 12, 16, 24, and 48 h. The serum was isolated from the blood by centrifugation for 10 min at 10,000 rpm. The serum samples were kept at -20 °C until quantification by HPLC analysis.

### 2.14 Drug quantification in plasma

A precise and accurate bioanalytical approach was devised for drug quantification in plasma samples. ACN was used to make 1 mg/ml stock solution of DL, and stock solution in ACN was used to make working standard solutions of 0.02, 0.1, 0.2, 0.5, 1, 2, 5, 10, 50, and 100 μg/ml. Overall, separate tubes containing 100 μg/ml of working solutions and 100 μg/ml of blank plasma were vortexed for 30 min. The tubes were then spun at 13,000 rpm for 5 min. The supernatant was collected, transferred to fresh tubes, and allowed to dry overnight at a temperature of 40°C. After that, the tubes were reconstituted with 100 μL of ACN, vortexed for 5 minutes, and 20 μL of supernatant was injected into HPLC to quantify DL. The working solution concentrations were 0.01, 0.05, 0.1, 0.25, 0.5, 1, 2.5, 5.0, 25.0, and 50.0 μg/ml. Low-quality control (LQC), medium quality control (MQC), and high-quality control (HQC) samples were created with different quantities of 0.005, 0.5, and 40 μg/ml, respectively. It was necessary to create quality control samples and triplicate calibrations for each concentration. A test tube containing 100 μL of test plasma and 100 μL of ACN was vortexed for 30 min to create the test samples. To assess the final outcomes, WinNonlin version 1.5.3 was used (Nisha et al., 2020a; Kumar, P., et al., 2018; Nisha et al., 2020b).

### 2.15 Bio-distribution studies

To assess the targeting potential, DL, DLF, and DLMF were exposed to albino Wistar rats for bio distribution study. The groups were divided into three categories: Group 1 DL: N-nitrosodiethylamine (DEN) +DL (DEN100 mg/kg, i. p. once a week for 6 weeks then DL 10 mg/kg given orally), Group 2 DLF: N-nitrosodiethylamine (DEN) +DLF (DEN100 mg/kg, i. p. once a week for 6 weeks then DLF 10 mg/kg given orally), Group 3 DLMF: N-nitrosodiethylamine (DEN) +DLMF (DEN100 mg/kg, i. p. once a week for 6 weeks then DLMF 10 mg/kg given orally). Following the euthanization of rats at certain time intervals, such as 4, 12, 24, and 48 h, different organs, including the kidney, lungs, heart, spleen, and liver, were removed and kept at -80 °C until processing the sample for drug quantification. Each organ was weighed separately and homogenized, and the amount of DL was quantified using the previously stated verified HPLC technique (Kumar, P., et al., 2018).

### 2.16 Molecular docking studies

To design the ligand (DL), the programs Chem-Draw Ultra 12.0 and Chem3D pro. 12.0 were used. The ligand was then modelled after the crystal structures of caspase-3 (1QX3) and 9 as found in the Protein Data Bank (http://www.rcsb.org/pdb) (2AR9). With the help of the visualizer included inside the BIOVIA Discovery Studio, the active site domain of the structure was located. Several previous publications were reposted; caspase-3 & 9 are essential proteins that play a role in the execution of apoptosis (Programmed Cell Death). Additionally, PDBQT files for ligand-free caspase-3 & 9 and ligand were produced using Auto Dock tools, version 1.5.6, followed by grid files. The interaction between caspase-3 and 9 proteins was then evaluated using Auto Dock tools, version 1.5.6 (Morris, G.M., et al., 1998; Khan, T., et al., 2018; Ni, C.Z., et al., 2003). The visualizer included in the BIOVIA Discovery Studio was used to assess the binding affinities in kcal/mol, the amino acids that were engaged in the interactions, and the hydrogen bonds (Chao, Y., et al., 2005; Kumar, P., et al., 2016; Agarwal, V., et al., 2021, D.S. BIOVIA, 2017).

### 2.17 Statistical data analysis

GraphPad Prism v.9.4.0 was used to perform the statistical analysis. The data were summarised by showing each variable’s mean and standard deviation (SD). The data was analyzed using a one-way ANOVA, which was followed by the Bonferroni multiple comparison test. *p*-value (***p < 0.001, **p < 0.01, and *p < 0.05) was judged statistically significant.

## 3 Results

**FIGURE 1:**
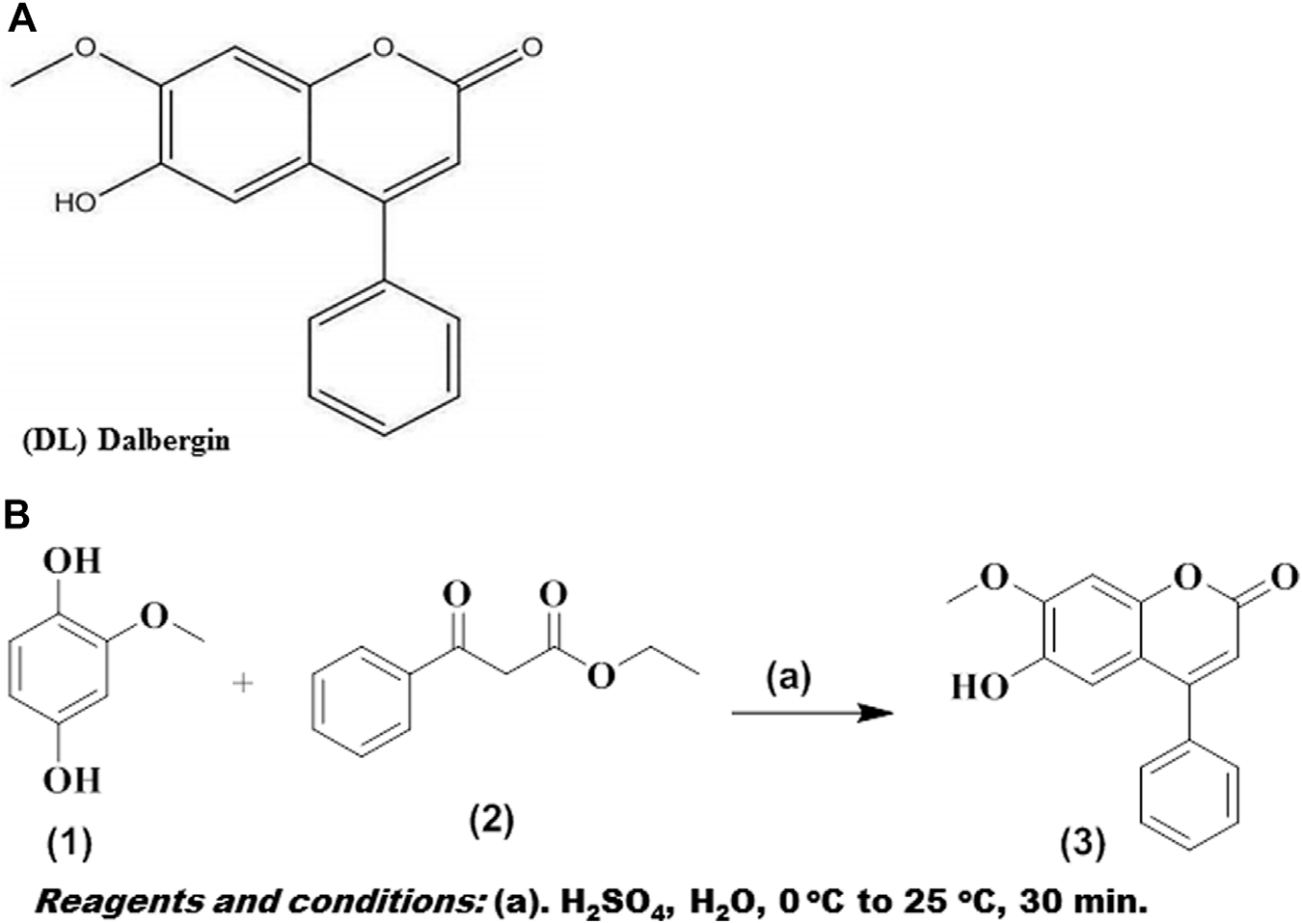
**(A)** Structure of dalbergin **(B)** Scheme.

The majority of primary liver cancer diagnoses occur in the last stages and are not made in the early stages (Charmantray, F. and Martelli, A., 2001). Despite their interesting cytotoxicity on diverse malignant cells, chemotherapy seldom causes damage to normal cells. (Zuco, V., et al., 2002). The structure of DL and the synthesis scheme was shown in Figure 1. DL is demonstrated here as an anticancer for the treatment of HCC. Low permeability and solubility of neoflavonoids result in decreased bioavailability (Perez-Sanchez, A., et al., 2017). Therefore, it is crucial to create a distribution mechanism that might enhance the solubilization of DL. As a consequence, the treatment window for DL was expanded by synthesising PLGA nanoformulations modified with galactose for targeting to ASGPR receptor in the liver tissues.

### 3.1 Formulation and optimization of DLF

Stability and physicochemical properties are crucial factors to consider while creating a novel medication delivery system. The DLF from the BBD in the current study confirmed that independent factors such as (X1) PLGA (X2) PVA (X3) sonication time had a significant impact on the dependent variables such as particle size and EE. A regression equation was developed for the dependent variables, R1 (PS) and R2 (% EE). The response surface graphs were used to describe the effect of the independent factors on the response variables and the interactions between them. The criteria that were chosen, in addition to their respective values and the optimization parameters, were explained (Table 1). The best parameters for the formulation of optimized DLF were 50 mg of polymer (PLGA), a concentration of 1% PVA, and a sonication time of 10 min. In terms of the end product (DLF), there was no significant deviation from the projected levels (Table 2). The particle size of the optimized DLF was determined to be 120.20 ± 1.82 nm, the PDI was 0.231 ± 0.01, and the EE was 94.67 ± 2.91% (Table 3).

**TABLE 1:** Independent variable and their levels in Box–Behnken design.

	Low	Medium	High
X1: PLGA (mg)	25	50	75
X2: PVA (%)	0.5	1.0	1.5
X3: Sonication time (min)	5	10	15
Coded values			
Dependent variables			
Y 1 = particle size (nm)			
Y 2 = entrapment efficiency (%)			

**TABLE 2:** Effect of various independent variables on dependent parameters for DL formulation prepared through Box−Behnken Design (all values expressed are Mean ± SD where *n* = 3).

Run no.	X1 PLGA (mg)	X2 PVA (%)	X sonication time (min)	Particle size (nm)	Entrapment efficiency (%)
1	50.00	1.50	5.00	128.0 ± 1.96	91.92 ± 1.84
2	25.00	1.00	15.00	150.60 ± 2.10	88.95 ± 2.12
3	50.00	0.50	15.00	135.20 ± 2.33	80.54 ± 2.02
4	25.00	1.00	5.00	185.90 ± 1.24	76.34 ± 1.09
5	50.00	0.50	5.00	159.70 ± 1.22	84.42 ± 2.01
6	75.00	1.50	10.00	178.90 ± 2.15	79.32 ± 3.22
7	25.00	0.50	15.00	162.50 ± 1.23	90.01 ± 2.10
**8**	**50.00**	**1.00**	**10.00**	**124.20 ± 1.44**	**92.01 ± 2.03**
9	75.00	1.50	5.00	172.80 ± 1.38	86.12 ± 2.11
10	75.00	0.50	10.00	145.40 ± 1.36	92.02 ± 2.88
11	50.00	1.00	10.00	131.20 ± 2.68	89.75 ± 1.57
12	50.00	1.00	10.00	159.80 ± 1.84	86.56 ± 1.42
13	25.00	1.50	5.00	179.90 ± 2.24	91.54 ± 1.78
14	50.00	0.50	15.00	152.30 ± 2.19	87.76.±1.33
15	50.00	0.50	15.00	155.10 ± 2.05	88.21 ± 1.22

Optimized value used in our study are indicated by bold.

**TABLE 3:** Predicted and observed values of particle size and % EE of the optimized formulation of DL.

Formulation	Composition			Response	
	PLGA (mg)	PVA (%)	Sonication time (min)	Particle size (nm)	Entrapment efficiency (%)
DLF (Predicted)	50.00	1.00	10.00	124.20 ± 1.99	92.04 ± 2.88
DLF (Observed)	50.00	1.00	10.00	120.20 ± 1.82	94.67 ± 2.91

### 3.2 Impact of DLF variables on the PS (Y1)

**FIGURE 2:**
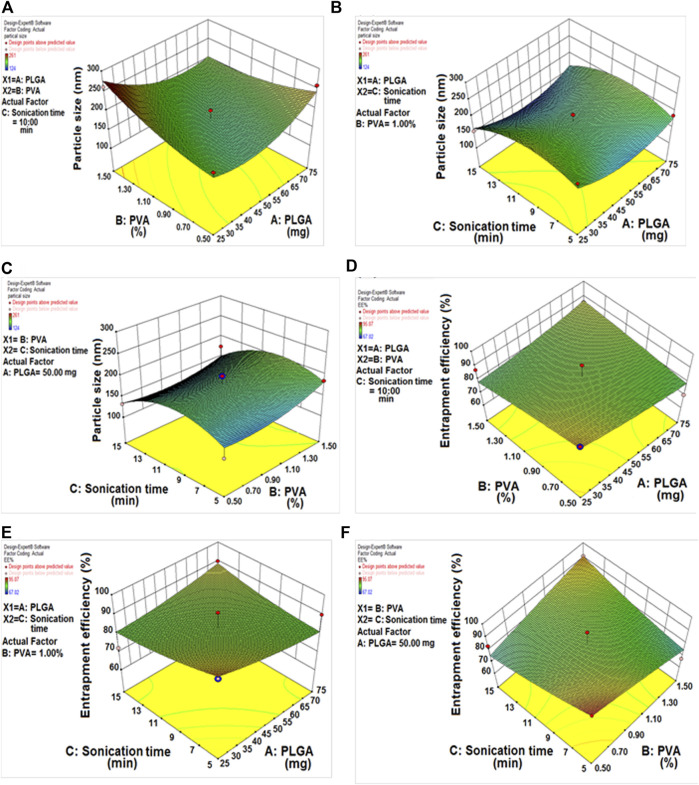
**(A)** Response 3D plot, effect of PLGA/PVA concentration on particle size. **(B)** Response 3D plot, effect of sonication time/PLGA concentration on particle size. **(C)** Response 3D plot, effect of sonication time/PVA concentration on particle size. **(D)** Response 3D plot, effect of PLGA/PVA concentration on entrapment efficiency. **(E)** Response 3D plot, effect of sonication time/PLGA concentration on entrapment efficiency. **(F)** Response 3D plot, effect of sonication time/PVA concentration on entrapment efficiency.

The DLF mean PS ranged from 124.20 ± 1.44 to 185.90 ± 1.24 nm. All formulations had a PDI of 0.4, indicating that the PS was consistent. Table 2 and Figures 2A–C show the influence of each variable on DLF PS, as calculated using the equation below.

Particle size (Y1)=+177.00+5.00×A+12.00×B−12.00×C+30.75×A×B−4.25×A×C−4.75×B×C

Where A denotes PLGA, B denotes PVA, and C denotes sonication time.

The positive sign (A and B) denotes a direct relationship between particle size and independent variables. In contrast, the negative sign (C) denotes an antagonistic relationship between the response and the factor. The concentrations of PLGA and PVA had a substantial beneficial impact on PS, the PS increased as the amount of PLGA and PVA increased. In the case of sonication time, the reverse effect was seen, with increased sonication time resulting in the formation of smaller nanoparticles.

### 3.3 Impact of PLGA-NPs variables on the % EE (Y2)

The effect of several different DLF variables on the EE is shown in Table 2 (the % EE varied from 76.34 ± 1.09 to 94.67 ± 2.91%), as well as in Figures 2D–F.

EE% (Y2)=+83.04+0.78×A+1.12×B+0.84×C+5.26×A×B+4.70×A×C+9.02×B×C

The concentrations of PLGA and encapsulation efficiency were found to have a positive relationship. Raising the PLGA content resulted in higher encapsulation efficiency values. In contrast, encapsulation efficiency decreased dramatically as PVA content increased. By extending the sonication period, the encapsulation efficiency was improved because the organic solvent diffuses rapidly in the aqueous phase (Tefas, L.R., et al., 2015).

### 3.4 Entrapment efficiency and drug loading

The observed values for the EE and drug loading of optimized DLF and DLMF were, respectively, 94.67 ± 2.91 and 13.42 ± 0.98% and 88.25 ± 1.34, and 11.36 ± 0.71%. Both of these measures showed a considerable drop due to the surface alteration.

### 3.5 *In vitro* drug release study

**FIGURE 3:**
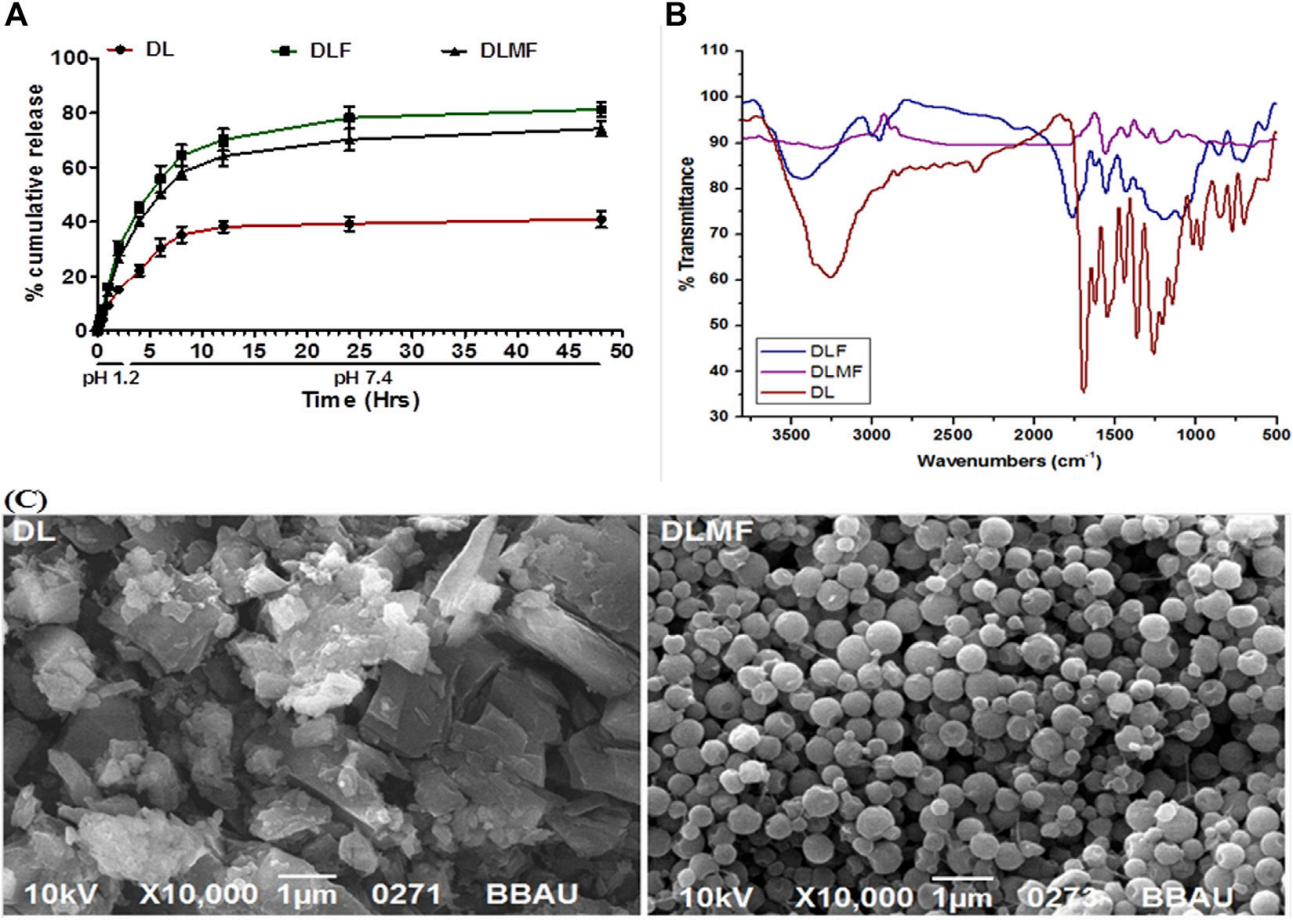
**(A)** In vitro release profile of DL, DLF and DLMF, Results are expressed as mean ± SD (*n* = 3). **(B)** FTIR spectrum of DL, DLF and DLMF. **(C)** SEM images of DL and DLMF.

Figure 3A shows the *in vitro* release evaluation of DL, DLF and DLMF. In the past, both DLF and DLMF had a release that was controlled in two phases. In both formulations (DLF and DLMF), the rate of drug release was found to be at its highest during the first 6 hours, after which it gradually decreased. The release of DLMF, on the other hand, was slower than that of DLF, which lasted up to 48 h. We observed around 40.97 ± 3.01, 81.24 ± 2.69, and 74.15 ± 2.81% cumulative DL, DLF and DLMF discharges up to 48 h. Surface modification decreases drug diffusion, a significant release mechanism in DLMF, which may explain the release. Furthermore, modified DLMF successfully achieved a sustained release of DL, which improved its targeting efficiency for treating HCC. This release pattern was beneficial because it elevated the creative release of DL, which sensitized HCC cells, thus increasing the effectiveness of the drug against HCC.

### 3.6 Drug interaction analysis using FTIR

DL, DLF, and DLMF showed O-H stretching mode at 2,900 cm^−1^ in FTIR testing. There was no interplay between DL and the polymers used when the C-O stretched between 1,625 and 1,650 cm^−1^. No typical interplay peaks existed in PLGA or PVA (Figure 3B).

### 3.7 Scanning electron microscopy studies

The final structure of DLMF particles was determined *via* SEM analysis of batch DL. After DLMF synthesis, DL’s rectangular particles morphed into spherical particles (Figure 3C).

### 3.8 Stability studies

After being stored at two distinct temperatures (4 ± 1°C and 25 ± 2°C), the DLMF that was synthesized was analyzed for its PS and its % EE to determine whether or not it remained stable at the intervals that were indicated. There were no indications of aggregate formation or phase separation in the optimized DLMF, which gave the impression of being an emulsion. After 90 days, descriptive statistics ((*p* < 0.05) revealed that there had been a rise in PS as well as a drop in the percentage of EE. As a direct consequence, the formulations exhibited a high degree of stability even after 3 months (Supplementary Table S1).

### 3.9 Evaluation of anticancer efficacy

**FIGURE 4:**
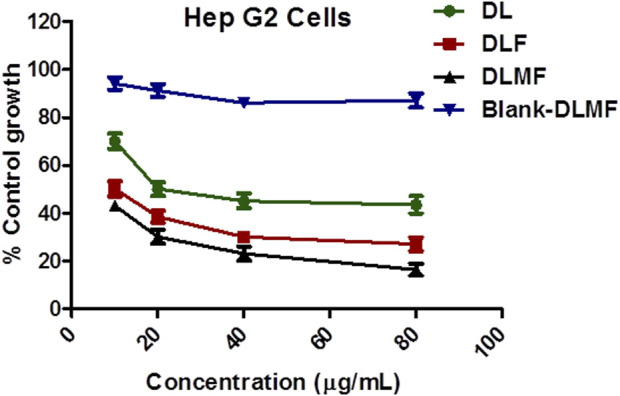
Growth curve of DL, DLF, and DLMF and Blank- DLMF on Hep G2 cells.

The HepG2 cell line was tested for cell cytotoxicity with pure DL, blank-DLMF, and DLMF at concentrations ranging from 10 to 80 μg/ml after 48 h (shown in Figure 4A). Our experimental results showed that the cytotoxicity of pure DL, DLF, and DLMF varied in a manner dependent on the dosage or the concentration but minimal cytotoxic potential activity for blank-DLMF. In HepG2 cells, DLMF showed more cytotoxic potential than pure DL. Pure DL (GI_50_ = 20 μg/ml) showed higher growth inhibitory concentration (GI_50_) than DL included in DLMF (GI_50_ < 10 μg/ml). This result shows that DLMF has more anticancer activity than DL. The higher cytotoxicity of DLMF is due to the impact of galactose targeting, which is attained by ASGPR targeting. Additionally, it produces a continuous release of DL in response to HepG2 cells absorbing it, eventually resulting in a significant quantity of cytotoxicity.

### 3.10 *In vitro* cellular internalization analysis

**FIGURE 5:**
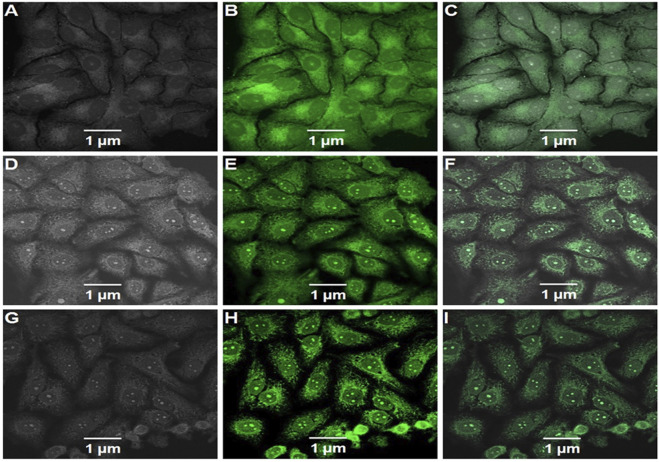
Confocal microscopic images of FITC-labeled DL **(A–C)**, FITC. labeled DLF **(D–F)**, and FITC labeled DLMF **(G–I)** showing the cellular uptake of nanoparticles in HepG2 cells. **(A,D,G)** Bright field, **(B,E,H)** green fluorescent channel and **(C,F,I)** overlay of channels.

This action was further demonstrated by confocal microscope pictures, which revealed that DLMF had more penetrability to HepG2 cells than DL and DLF, suggesting increased cellular absorption of DLMF after 2 h of incubation (Figure 5).

### 3.11 HPLC determination of DL, DLF, and DLMF plasma concentrations

**FIGURE 6:**
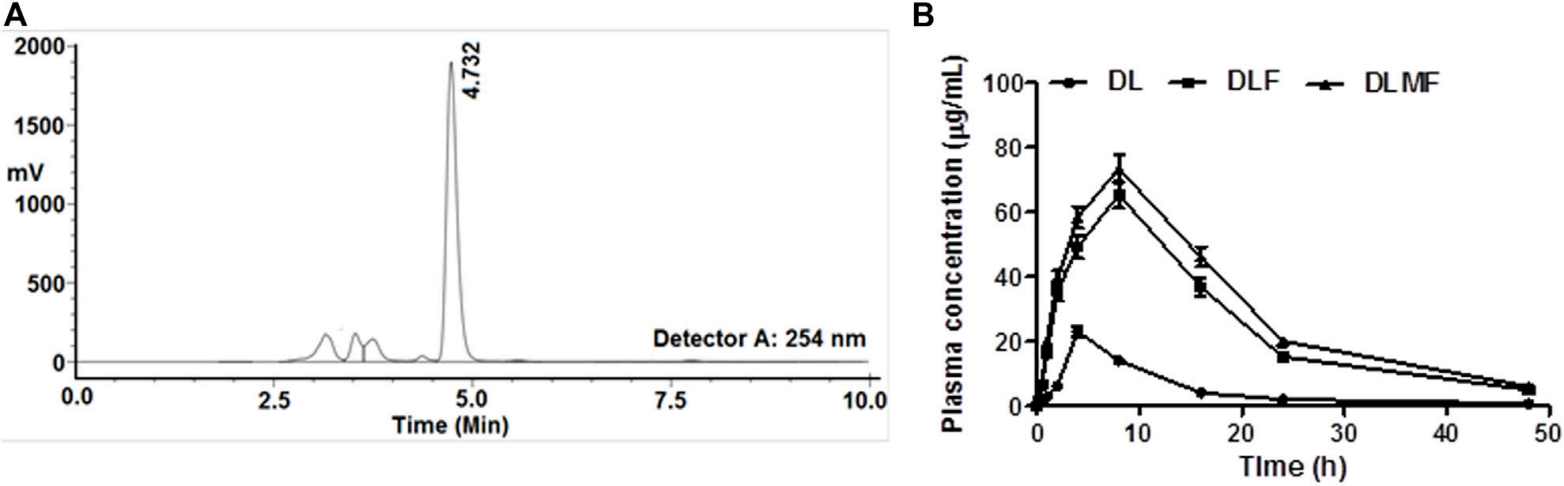
**(A)** Chromatogram of DL (RT 4.732 min) through HPLC, **(B)** Plasma drug concentrations at various time points after oral administration of DL, DLF and DLMF in albino Wistar rats.

The total run time was 10 min as shown in the chromatogram, and the retention time (RT) for DL was calculated to be 4.732 min (Figure 6A). The correlation coefficient (r^2^) for DL came out to be 0.9901 when it was analyzed using linear regression with a range of 0.01–50 μg/ml. It was determined that the test had an accuracy of between 83 and 94%, and the sample recovery rate ranged from 80 to 94%. The following is a description of the maximum plasma concentration (C_max_) and the length of time needed to reach the highest plasma concentration (T_max_), which can be found in Table 4 and Figure 6B, respectively: 23.14 μg/ml, 65.33 μg/ml, and 4 h for DL, DLF; 73.58 μg/ml, and 8 h for DLMF. At 9.63, 11.02, and 12.09 h, the plasma concentrations of DL, DLF, and DLMF reached 50% (T_1/2_).

**TABLE 4:** Pharmacokinetic parameters of DL, DLF and DLMF in rat plasma.

Parameter	DL	DLF	DLMF
t_1/2_	9.63	11.02	12.09
T_max_(h)	4.00	8.00	8.00
C_max_ (µg/ml)	23.14 ± 0.84	65.33 ± 1.25	73.58 ± 1.31
AUMC (µg.h^2^/ml)	2806.95	17,420.45	21,558.00
MRT (h)	11.50 ± 1.01	14.38 ± 1.22	14.75 ± 1.23
CL (h)	0.002	0.0013	0.0011
AUC_0-∞_ (µg.h/ml)	243.91 ± 10.27	1211.22 ± 23.39	1460.67 ± 29.14

**Abbreviations:** t_1/2_, time required to reach 50% plasma concentration; Tmax, time required to reach the maximum plasma concentration; *C*max, maximum plasma concentration; AUMC, area under the first moment curve; MRT, mean residence time; CL, clearance; AUC, area under the curve.

### 3.12 Biodistribution study

**FIGURE 7:**
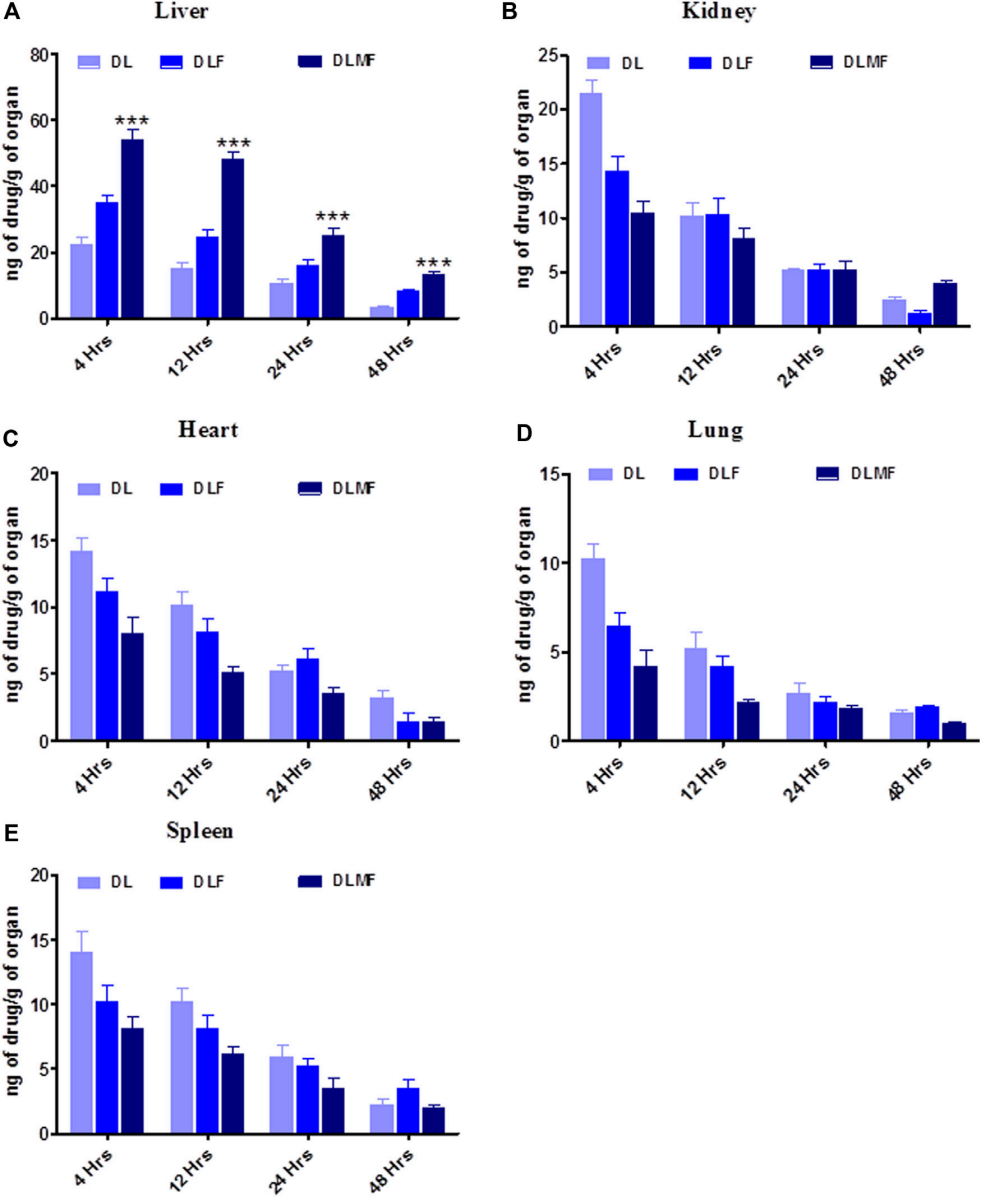
Bio-distribution of DL, DLF and DLMF In various organs. **(A–E)** are showed individual organ like, **(A)** Liver, **(B)** Kidney, **(C)** Heart, **(D)** Lung, **(E)** Spleen.

A delivery method with more excellent capability for targeting cancer is required to meet the criteria for a successful targeted anticancer therapy. As a consequence, research on the biodistribution of DLMF was carried out to ascertain the effectiveness of its targeting. A comparison of the distribution of DL throughout the body’s several organs may be seen in this image (Figure 7). According to the research findings, rats administered with DLMF showed a higher concentration of DL in the tumor tissue compared to the animals received DLF or pristine DL. After oral administration of DLMF, the drug accumulation in tumor tissues was significantly increased (*p* < 0.001), with 54.23 ± 3.02 ng/g after 4 h and 48.19 ± 2.17 ng/g at 12 h, respectively. After oral administration of DLF, however, low amounts of DL remained in the tumor organ after 4 h (35.14 ± 2.15 ng/g), although these concentrations reduced significantly at 12 h (24.52 ± 2.41 ng/g).

### 3.13 Molecular docking studies

**FIGURE 8:**
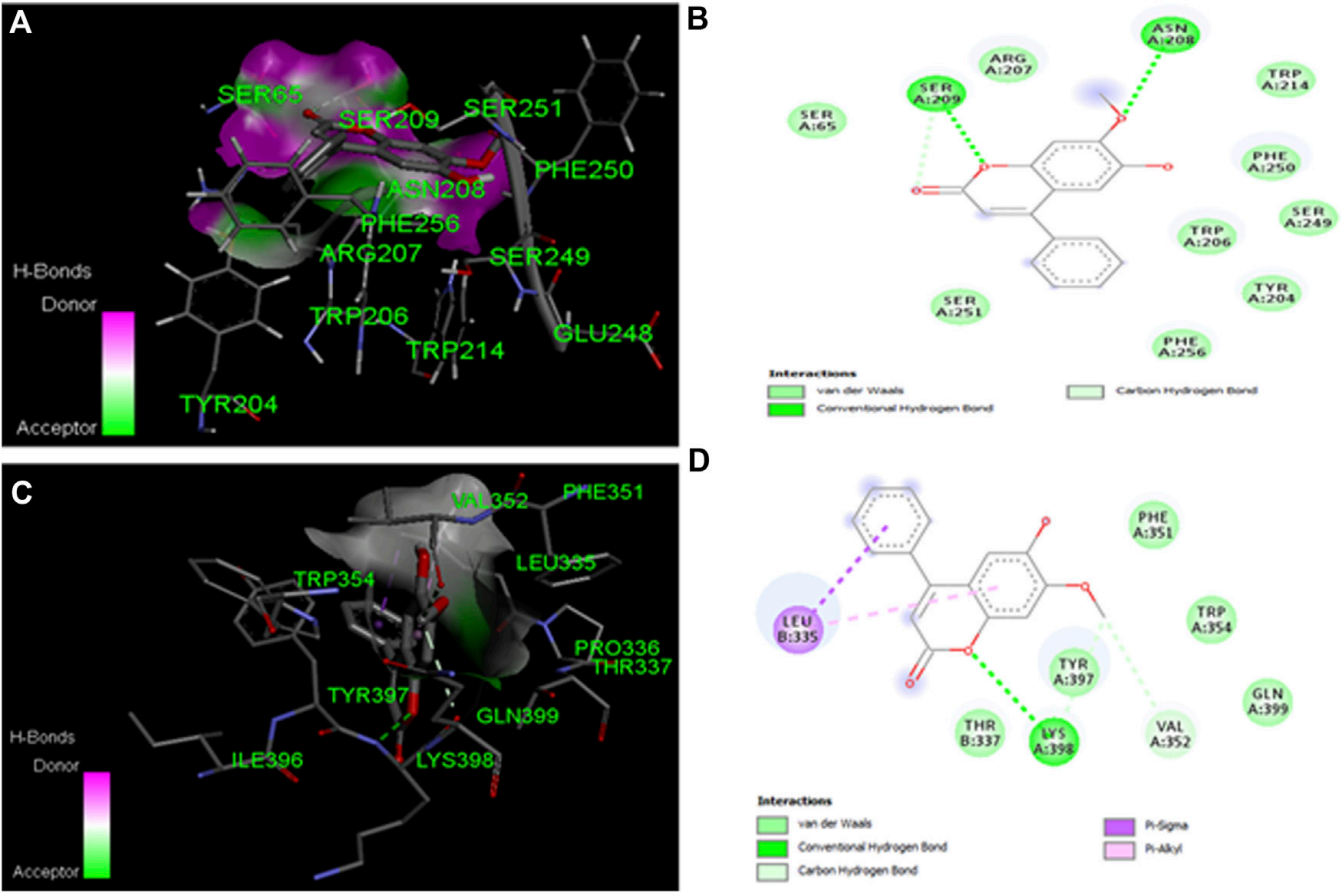
Molecular docking studies of dalbergin with caspage-3 and 9.**(A)** The output of AutoDock 3D diagram shows the binding site residues of caspase-3 with the dalbergin, with binding affinity of −6.7 kcal/mol. **(B)** The 2D structure exhibits different types of interactions formed between caspase-3 and dalbergin. The dark green and purple dotted lines indicate H-bond interactions between caspage-3. **(C)** The output of AutoDock 3D diagram shows the binding site residues of caspase-9 with the dalbergin, with binding affinity of −6.6 kcal/mol. **(D)** The 21:1 structure exhibits different types of interactions formed between caspase-9 and dalbergin. The dark green and purple dotted lines indicate H-bond and Pi-Sigma interactions between caspage-9 and dalbergin.

The ligand (DL) molecular docking experiments for caspase-3 and 9 interaction docking investigations reveal binding interactions between proteins and respective ligands. Ligand binding characteristics with caspase-3 and 9 were determined using the molecular docking program Auto Dock tools. According to Auto Dock tools, the binding affinity of the ligand to caspase-3 was −6.7 kcal/mol, while the binding affinity of the ligand to caspase-9 was −6.6 kcal/mol. The two hydrogen bonds are the primary reason for the caspase-3 binding affinities. SER 65, TYR 204, TRP 206, ARG 207, ASN 208, SER 209, TRP 214, SER 249, PHE 250, SER 251, and PHE 256 are some of the amino acids involved in the interaction (Table 5). The binding affinities of caspase-9 were ascribed to a single hydrogen bond with Pi sigma. LEU 335, THR 337, PHE 351, VAL 352, TRP 354, TYR 357, LYS 398, and GLN 399 are some amino acids involved in the interaction (Table 5). The ligand, caspase-3 and 9, interaction are shown in Figure 8.

**TABLE 5:** *In-silico* study of DL with caspases.

S.N.	Proteins/PDB code	Amino acids involved in interactions	Affinity (kcal/mol)	H-bonds
1	Caspase-3 (1QX3)	SER 65, TYR 204, TRP 206, ARG 207, ASN 208, SER 209, TRP 214, SER 249, PHE 250, SER 251, PHE 256	−6.7	2
2	Caspase-9 (2AR9)	LEU 335, THR 337, PHE 351, VAL 352, TRP 354, TYR 357, LYS 398, GLN 399	−6.6	1

## 4 Discussion

The liver is an extremely important organ in the human body. In this organ, it gets infection by viruses and bacteria, which leads to cirrhosis and is converted to primary liver cancer, known as HCC (Wang, R., et al., 2021). Primary liver cancer is not identified in the early stage but is mostly identified in the last stage (Pons, F., et al., 2005). Chemotherapeutics have sparked a lot of interest in anticancer drug discovery because of their high cytotoxicity (Charmantray, F. and Martelli, A., 2001). Chemotherapeutics seldom harm normal cells, despite their fascinating cytotoxicity on various malignant cells. A comparison of the results of many tests using different cell lines revealed that the cancer cells are much more resistant to chemotherapy than normal cells of various origins, demonstrating the tumor selectivity of the treatment (Zuco, V., et al., 2002). Herein, DL is shown as an anticancer for treating HCC, and it is the neoflavonoids category of drugs. According to the biopharmaceutics categorization system, neoflavonoids are classified as BCS class IV substances. The neoflavonoids have low solubility, and low permeability lead to less bioavailability (Perez-Sanchez, A., et al., 2017). Therefore, developing a delivery system that may improve DL’s solubilization is essential. As a result, synthesized PLGA nanoformulations modified with galactose for targeting to ASGPR receptor in the liver tissues increased the therapeutic window of DL. However, as an anticancer agent, DL suffers from several significant drawbacks, the most notable of which are drug resistance, non-homogeneous systemic availability, low bioavailability, lowest therapeutic effectiveness, and dose-related toxicity (Xiao-qiang WA, et al., 2009). The therapeutic value of DL is diminished as a result of these restrictions. As a result, we created and improved DLMF to increase the therapeutic effectiveness, bioavailability, and specificity of DL, while reducing the dosage and improving tissue selectivity of the drug. DLMF has been shown to have higher anticancer efficacy, and we have investigated the mechanism underlying this potential at the cellular level (Mahdizade Valojerdi F, et al., 2022). The creation of DL nanoformulation for the treatment of HCC was attempted in the present study, and it was successful.

QbD was utilized to generate an optimum formulation that took the use of a variety of characteristics. Based on the response surface plot, it was discovered that increasing the concentrations of PLGA and PVA had a significant impact on PS. The PS was increasing when increasing the quantity of PLGA and PVA. In the case of sonication duration, the reverse effect was seen, with increased sonication time resulting in the creation of smaller particles (Vandervoort, J. and Ludwig, A., 2002). In the past, andrographolide nanoparticles have been produced using the natural polymers PLGA and PVA, which are both safe to use (Roy, P., et al., 2010). PVA, a biopolymer, was used to stabilise the nanoparticles and reduce the interfacial tension, both of which contributed to improving the particles’ overall stability. The impact of PVA on stability has been previously documented, and a similar approach was used in our investigation (Murakami, H., et al., 1997). It was shown that 50 mg of polymer (PLGA) and 1% PVA concentration, together with a sonication time of 10 min, were the most effective conditions for forming PLGA nanoparticles. Neither the final result nor the expected values differed significantly from their predictions (DLMF). They had an average particle size of 120.20 ± 1.83 nm, PDI of 0.231, and an average percent efficiency (EE) of 94.67%. The FTIR measurements showed no interaction between DL and the co-polymers utilized.

DLF and DLMF formulations were shown to be releasing drugs in a regulated way *in vitro* drug release experiments, which is advantageous for sustained action (Amirsaadat, S., et al., 2021; Vijayakumar, M.R., et al., 2016).

The stability analysis of DLMF at 25 ± 2 and 4 ± 1°C for 90 days found no statistically significant differences in PS and EE. As a result, the stability test revealed that the DLMF formulation is stable for the DL system. The combination of PLGA and the capacity of PVA to shield medicines from chemical instability can be credited for the NPs’ remarkable physical stability (Nisha et al., 2020b; Abdelmoneem, M.A., et al., 2019). Later, this method was repeated on a bigger measurer, and a greater quantity of DLMF was synthesized for evaluation and *in vitro* and *in vivo* experiments, as performed as previously with other components (Kumar et al., 2018).

According to study findings, coating the surface of nanoparticles with hydrophilic polymers resulted in increased cellular absorption. As a result of their functionalization, DLMF have longer blood circulation duration and a reduced RES absorption, guaranteeing that they are taken up by cancer cells that have an overexpressed ASGPR. EPR effect is also reported in achieving the higer cell accumulation (Nisha et al., 2020a). As an ASGPR ligand, Galactose actively targets HCC cells, which have ASGPR overexpression *via* endocytosis (Gupta, S., et al., 2013).

To begin with, the drugs DL and DLMF were tested on HepG2 cell lines using the standard medication. DLF showed good selectivity for the HepG2 cell line (GI_50_ = 10 μg/ml). However, the pristine DL showed considerable cytotoxicity (GI_50_ = 20 μg/ml) against the same cell line. The *in vitro* experiments on DL-containing PLGA nanoparticles revealed that they were effective against malignant cells, proving their importance for the delivery of anticancer activity. For some reason, when DLMF were used in *in vitro* experiments, the growth curve showed that the control growth rates were 50% at <10 μg/ml concentration but that they were not considered to be in the negative zone of percent control growth rate (Kumar, P., et al., 2018; Liu and Luo, 2012). As a result, it is reasonable to predict that DLMF will have a tremendous ability to destroy cancerous cells while the simultaneously lower rate of mortality experienced by normal cells. In addition, this activity was further validated by confocal microscopy, which demonstrated that DLMF has a potent penetrating ability and could penetrate the HepG2 cell line to a greater extent than the parent DL compound (Bharali DJ, et al., 2011). According to the results of these two trials, DLMF demonstrated more antineoplastic feasible than pure DL.

In addition, it is required to evaluate the *in vivo* pharmacokinetics of produced DL, DLF, and DLMF following oral administration of the compounds. DLMF had greater distribution in plasma than the pure drug. It is possible that the smaller PS of DLMF, which, upon oral administration of the chemical, was subsequently absorbed to a greater degree, causes this effect. It was proven by the AUC data obtained from the pharmacokinetic investigations that DLMF had about four-fold more excellent plasma distribution than the parent compound. Biodistribution tests further support the idea that DLMF has the ability to target the accumulating hepatic location while restricting entry into other necessary organs. Specific affinities and ASGPR binding on HCC cells may be used to carry out this experiment (Roufogalis B. 2012).

According to the findings of the study, hydrophilic polymers are utilized to functionalize the surfaces of nanoparticles, regardless of size, the particle’s cellular absorption. The functionalization of these nanoparticles causes prolonged blood circulation time and low RES absorption, increasing the possibility that they may be absorbed by cancer cells that have overexpressed the DLMF, and so DLMF may provide a unique therapeutic option for HCC (Kashyap D, et al., 2021).

The apoptotic signaling proteins such as caspase-3 and 9 are known to be associated with HCC, which has a role in the program cell death (apoptosis) (Kattan, S.W., et al., 2020). Many previous publications reported the strong binding affinities of caspase-3 & 9 with ligand to lead apoptosis in cancer cells (Kirubhanand, C., et al., 2020). Herein, we used DL to undertake molecular docking experiments on caspase-3 and 9 targets. Their hydrogen bonding potential and binding affinities (kcal/mol) were determined. Docking pictures of drugs, including amino acids implicated in binding postures, are shown in Figure 8. High binding affinities to DL were observed for both caspase-3 (−6.7 kcal/mol and 2 H-bonds) and caspase-9 (−6.6 kcal/mol and 1 H-bond), which is suggestive of anticancer actions for both proteins (Wanandi, S.I., et al., 2020).

## 5 Conclusion

A novel formulation of DLMF was effectively created, and the formulation was characterized by morphological and physiological parameters. The *in vitro* anti-HCC effects of the DLMF were discovered and confirmed by the cytotoxicity and cellular uptake study against HepG2 Cells. *In-vivo* oral pharmacokinetics, as well as the biodistribution effects of the compound, were discovered and confirmed. The plasma t_1/2_, C_max_, and T_max_ of DLMF were greater than those of the pristine DL. DLMF was shown to be more excellent and effective biodistribution in the hepatic tissue among all vital organs. It is possible that this investigation’s findings will prove the preventative activity of DLMF against HCC. Furthermore, the affinity of drug with caspase 3 and 9 proteins in *in-silico* study were found to be -6.7 kcal/mol and -6.6 kcal/mol, respectively. Our *in-silico* analysis revealed that DL has strong apoptotic properties against HCC. As an outcome, the research may confirm that DLMF may be used effectively in the treatment of HCC.

## Data Availability

The original contributions presented in the study are included in the article/Supplementary Material, further inquiries can be directed to the corresponding author.

## References

[B1] Abdelmoneem M. A. Elnaggar M. A. Hammady R. S. Kamel S. M. Helmy M. W. Abdulkader M. A. (2019). Dual-targeted lactoferrin shell-oily core nanocapsules for synergistic targeted/herbal therapy of hepatocellular carcinoma. ACS Appl. Mat. Interfaces 11 (30), 26731–26744. 10.1021/acsami.9b10164 31268657

[B2] Acharya S. K. (2014). Epidemiology of hepatocellular carcinoma in India. J. Clin. Exp. Hepatol. 4, S27–S33. 10.1016/j.jceh.2014.05.013 PMC4284206 25755607

[B3] Adlravan E. Nejati K. Karimi M. A. Mousazadeh H. Abbasi A. Dadashpour M. (2021). Potential activity of free and PLGA/PEG nanoencapsulated nasturtium officinale extract in inducing cytotoxicity and apoptosis in human lung carcinoma A549 cells. J. Drug Deliv. Sci. Technol. 61, 102256. 10.1016/j.jddst.2020.102256

[B4] Agarwal V. Kaushik A. S. Rehman M. Chaudhary R. Jawaid T. Kamal M. (2021). Interleukin-6 expression and its modulation by diacerein in a rat model of chronic stress induced cardiac dysfunction. Heliyon 7 (12), e08522. 10.1016/j.heliyon.2021.e08522 34917808 PMC8665349

[B5] Al-Salama Z. T. Syed Y. Y. Scott L. J. (2019). Lenvatinib: A review in hepatocellular carcinoma. Drugs 79 (6), 665–674. 10.1007/s40265-019-01116-x 30993651

[B6] Amirsaadat S. Jafari-Gharabaghlou D. Alijani S. Mousazadeh H. Dadashpour M. Zarghami N. (2021). Metformin and Silibinin co-loaded PLGA-PEG nanoparticles for effective combination therapy against human breast cancer cells. J. Drug Deliv. Sci. Technol. 61, 102107. 10.1016/j.jddst.2020.102107

[B7] Amjadi I. Rabiee M. Hosseini M. S. (2013). Anticancer activity of nanoparticles based on PLGA and its co-polymer: *In-vitro* evaluation. Iran. J. Pharm. Res. 12 (4), 623–634. 24523742 PMC3920687

[B8] Bakrania A. Zheng G. Bhat M. (2021). Nanomedicine in hepatocellular carcinoma: A new frontier in targeted cancer treatment. Pharmaceutics 14 (1), 41. 10.3390/pharmaceutics14010041 35056937 PMC8779722

[B9] Bharali D. J. Siddiqui I. A. Adhami V. M. Chamcheu J. C. Aldahmash A. M. Mukhtar H. (2011). Nanoparticle delivery of natural products in the prevention and treatment of cancers: Current status and future prospects. Cancers 3 (4), 4024–4045. 10.3390/cancers3044024 24213123 PMC3763408

[B10] Biovia D. S. (2017). Discovery studio modeling environment. San Diego: Dassault Systèmes.

[B11] Bteich F. Di Bisceglie A. M. (2019). Current and future systemic therapies for hepatocellular carcinoma. Gastroenterol. Hepatol. 15 (5), 266–272. PMC6589844 31360140

[B12] Chao Y. Shiozaki E. N. Srinivasula S. M. Rigotti D. J. Fairman R. Shi Y. (2005). Engineering a dimeric caspase-9: A re-evaluation of the induced proximity model for caspase activation. PLoS Biol. 3 (6), e183. 10.1371/journal.pbio.0030183 15941357 PMC1088972

[B13] Charmantray F. Martelli A. (2001). Interest of acridine derivatives in the anticancer chemotherapy. Curr. Pharm. Des. 7 (17), 1703–1724. 10.2174/1381612013397131 11562307

[B14] Chen Z. Xie H. Hu M. Huang T. Hu Y. Sang N. (2020). Recent progress in treatment of hepatocellular carcinoma. Am. J. Cancer Res. 10 (9), 2993–3036. 33042631 PMC7539784

[B15] D'souza A. A. Devarajan P. V. (2015). Asialoglycoprotein receptor mediated hepatocyte targeting—strategies and applications. J. Control. Release 203, 126–139. 10.1016/j.jconrel.2015.02.022 25701309

[B16] Danhier F. Ansorena E. Silva J. M. Coco R. Le Breton A. Préat V. (2012). PLGA-Based nanoparticles: An overview of biomedical applications. J. Control. Release 161 (2), 505–522. 10.1016/j.jconrel.2012.01.043 22353619

[B17] Gupta S. Agarwal A. Gupta N. K. Saraogi G. Agrawal H. Agrawal G. P. (2013). Galactose decorated PLGA nanoparticles for hepatic delivery of acyclovir. Drug Dev. Ind. Pharm. 39 (12), 1866–1873. 10.3109/03639045.2012.662510 22397550

[B18] Huang K. W. Lai Y. T. Chern G. J. Huang S. F. Tsai C. L. Sung Y. C. (2018). Galactose derivative-modified nanoparticles for efficient siRNA delivery to hepatocellular carcinoma. Biomacromolecules 19 (6), 2330–2339. 10.1021/acs.biomac.8b00358 29808997

[B19] Javan N. Khadem Ansari M. H. Dadashpour M. Khojastehfard M. Bastami M. Rahmati-Yamchi M. (2019). Synergistic antiproliferative effects of co-nanoencapsulated curcumin and chrysin on mda-mb-231 breast cancer cells through upregulating mir-132 and mir-502c. Nutr. Cancer 71 (7), 1201–1213. 10.1080/01635581.2019.1599968 30955355

[B20] Kanoujia J. Faizan M. Parashar P. Singh N. Saraf S. A. (2021). Curcumin loaded sericin nanoparticles: Assessment for biomedical application. Food Hydrocoll. Health 1, 100029. 10.1016/j.fhfh.2021.100029

[B21] Kashyap D. Tuli H. S. Yerer M. B. Sharma A. Sak K. Srivastava S. (2021). Natural product-based nanoformulations for cancer therapy: Opportunities and challenges. Semin. Cancer Biol. 69, 5–23. 10.1016/j.semcancer.2019.08.014 31421264

[B22] Kattan S. W. Nafie M. S. Elmgeed G. A. Alelwani W. Badar M. Tantawy M. A. (2020). Molecular docking, anti-proliferative activity and induction of apoptosis in human liver cancer cells treated with androstane derivatives: Implication of PI3K/AKT/mTOR pathway. J. Steroid Biochem. Mol. Biol. 198, 105604. 10.1016/j.jsbmb.2020.105604 31982513

[B23] Kawase M. Sakagami H. Motohashi N. Hauer H. Chatterjee S. S. Spengler G. (2005). Coumarin derivatives with tumor-specific cytotoxicity and multidrug resistance reversal activity. vivo 19 (4), 705–711. 15999537

[B24] Khan T. Lawrence A. J. Azad I. Raza S. Khan A. R. (2018). Molecular docking simulation with special reference to flexible docking approach. JSM Chem. 6 (1), 1053–1057.

[B25] Kirubhanand C. Selvaraj J. Rekha U. V. Vishnupriya V. Nalini D. Mohan S. K. (2020). Molecular docking data of piperine with bax, caspase 3, cox 2 and caspase 9. Bioinformation 16 (6), 458–461. 10.6026/97320630016458 32884209 PMC7452745

[B26] Kumar P. Kushwaha P. Ahmad N. Maurya S. W. Dev K. Khedgikar V. (2017). Design and synthesis of dalbergin analogues and evaluation of anti-osteoporotic activity. Bioorg. Med. Chem. Lett. 27 (8), 1765–1775. 10.1016/j.bmcl.2017.02.062 28274632

[B27] Kumar P. Rawat A. Keshari A. K. Singh A. K. Maity S. De A. (2016). Anti-proliferative effect of isolated isoquinoline alkaloid from Mucuna pruriens seeds in hepatic carcinoma cells. Nat. Prod. Res. 30 (4), 460–463. 10.1080/14786419.2015.1020489 25774560

[B28] Kumar P. Singh A. K. Raj V. Rai A. Keshari A. K. Kumar D. (2018). Poly (lactic-co-glycolic acid)-loaded nanoparticles of betulinic acid for improved treatment of hepatic cancer: Characterization, *in vitro* and *in vivo* evaluations. Int. J. Nanomedicine 13, 975–990. 10.2147/IJN.S157391 29497292 PMC5818879

[B29] Li Y. Huang G. Diakur J. Wiebe L. I. (2008). Targeted delivery of macromolecular drugs: Asialoglycoprotein receptor (ASGPR) expression by selected hepatoma cell lines used in antiviral drug development. Curr. Drug Deliv. 5 (4), 299–302. 10.2174/156720108785915069 18855599

[B30] Li Y. Martin R. C. (2011). Herbal medicine and hepatocellular carcinoma: Applications and challenges. Evid. Based. Complement. Altern. Med. 2011, 541209. 10.1093/ecam/neq044 PMC3140057 21799681

[B31] Liu H. Zhang C. X. Ma Y. He H. W. Wang J. P. Shao R. G. (2016). SphK1 inhibitor SKI II inhibits the proliferation of human hepatoma HepG2 cells via the Wnt5A/β-catenin signaling pathway. Life Sci. 151, 23–29. 10.1016/j.lfs.2016.02.098 26944438

[B32] Liu Y. Luo W. (2012). Betulinic acid induces Bax/Bak-independent cytochrome c release in human nasopharyngeal carcinoma cells. Mol. Cells 33 (5), 517–524. 10.1007/s10059-012-0022-5 22526391 PMC3887732

[B33] Llovet J. M. Ricci S. Mazzaferro V. Hilgard P. Gane E. Blanc J. F. (2008). Sorafenib in advanced hepatocellular carcinoma. N. Engl. J. Med. 359 (4), 378–390. 10.1056/NEJMoa0708857 18650514

[B34] Ly H. T. Truong T. M. Nguyen T. T. H. Nguyen H. D. Zhao Y. Le V. M. (2021). Phytochemical screening and anticancer activity of the aerial parts extract of *Xanthium strumarium* L. on HepG2 cancer cell line. Clin. Phytosci. 7 (1), 14–18. 10.1186/s40816-021-00252-w

[B35] Mahdizade Valojerdi F. Goliaei B. Rezakhani N. Nikoofar A. Keshmiri Neghab H. Soheilifar M. H. (2022). *In vitro* radiosensitization of T47D and MDA-MB-231 breast cancer cells with the neoflavonoid dalbergin. Middle East J. Cancer. 10.30476/mejc.2022.91913.1637

[B36] Mahdizade V. F. Goliaei B. Parivar K. Nikoofar A. (2019). Effect of a neoflavonoid (dalbergin) on T47D breast cancer cell line and mRNA levels of p53, bcl-2, and STAT3 genes. Iran. Red. Crescent Med. J. 21, e87175. 10.5812/ircmj.87175

[B37] Makadia H. K. Siegel S. J. (2011). Poly lactic-co-glycolic acid (PLGA) as biodegradable controlled drug delivery carrier. Polymers 3 (3), 1377–1397. 10.3390/polym3031377 22577513 PMC3347861

[B38] Morris G. M. Goodsell D. S. Halliday R. S. Huey R. Hart W. E. Belew R. K. (1998). Automated docking using a Lamarckian genetic algorithm and an empirical binding free energy function. J. Comput. Chem. 19 (14), 1639–1662. 10.1002/(sici)1096-987x(19981115)19:14<1639:aid-jcc10>3.0.co;2-b

[B39] Murakami H. Kawashima Y. Niwa T. Hino T. Takeuchi H. Kobayashi M. (1997). Preparation of poly(DL-lactide-co-glycolide) nanoparticles by modified spontaneous emulsification solvent diffusion method. Int. J. Pharm. 149 (1), 143–152. 10.1016/s0378-5173(99)00187-8 10502620

[B40] Nair A. B. Shah J. Al-Dhubiab B. E. Patel S. S. Morsy M. A. Patel V. (2019). Development of asialoglycoprotein receptor-targeted nanoparticles for selective delivery of gemcitabine to hepatocellular carcinoma. Molecules 24 (24), 4566. 10.3390/molecules24244566 PMC6943439 31847085

[B41] Ni C. Z. Li C. Wu J. C. Spada A. P. Ely K. R. (2003). Conformational restrictions in the active site of unliganded human caspase‐3. J. Mol. Recognit. 16 (3), 121–124. 10.1002/jmr.615 12833566

[B42] Nisha R. Kumar P. Gautam A. K. Bera H. Bhattacharya B. Parashar P. (2020a). Assessments of *in vitro* and *in vivo* antineoplastic potentials of β-sitosterol-loaded PEGylatedniosomes against hepatocellular carcinoma. J. Liposome Res. 31, 304–315. 10.1080/08982104.2020.1820520 32901571

[B43] Nisha R. Kumar P. Kumar U. Mishra N. Maurya P. Singh S. (2020b). Fabrication of imatinib mesylate-loaded lactoferrin-modified PEGylated liquid crystalline nanoparticles for mitochondrial-dependent apoptosis in hepatocellular carcinoma. Mol. Pharm. 18 (3), 1102–1120. 10.1021/acs.molpharmaceut.0c01024 33356314

[B44] Perez-Sanchez A. Borras-Linares I. Barrajon-Catalan E. Arraez-Roman D. Gonzalez-Alvarez I. Ibanez E. (2017). Evaluation of the intestinal permeability of rosemary (Rosmarinus officinalis L.) extract polyphenols and terpenoids in Caco-2 cell monolayers. PLoS One 12 (2), e0172063. 10.1371/journal.pone.0172063 28234919 PMC5325326

[B45] Pons F. Varela M. Llovet J. M. (2005). Staging systems in hepatocellular carcinoma. Hpb 7 (1), 35–41. 10.1080/13651820410024058 18333159 PMC2023920

[B46] Rimassa L. (2018). Drugs in development for hepatocellular carcinoma. Gastroenterol. Hepatol. 14 (9), 542–544. PMC6194654 30364332

[B47] Roufogalis B. (2012). Flavonoid pharmacokinetics: Methods of analysis, preclinical and clinical pharmacokinetics, safety, and toxicology. New Jersey, United States: John Wiley & Sons.

[B48] Roy P. Das S. Bera T. Mondol S. Mukherjee A. (2010). Andrographolide nanoparticles in leishmaniasis: Characterization and *in vitro* evaluations. Int. J. Nanomedicine 5, 1113–1121. 10.2147/IJN.S14787 21270962 PMC3023240

[B49] Samadzadeh S. Mousazadeh H. Ghareghomi S. Dadashpour M. Babazadeh M. Zarghami N. (2021). *In vitro* anticancer efficacy of Metformin-loaded PLGA nanofibers towards the post-surgical therapy of lung cancer. J. Drug Deliv. Sci. Technol. 61, 102318. 10.1016/j.jddst.2020.102318

[B50] Song X. Wang J. Xu Y. Shao H. Gu J. (2019). Surface-modified PLGA nanoparticles with PEG/LA-chitosan for targeted delivery of arsenic trioxide for liver cancer treatment: Inhibition effects enhanced and side effects reduced. Colloids Surf. B Biointerfaces 180, 110–117. 10.1016/j.colsurfb.2019.04.036 31030022

[B51] Song X. You J. Shao H. Yan C. (2018). Effects of surface modification of As2O3-loaded PLGA nanoparticles on its anti-liver cancer ability: An *in vitro* and *in vivo* study. Colloids Surf. B Biointerfaces 169, 289–297. 10.1016/j.colsurfb.2018.05.024 29793091

[B52] Tefas L. R. Tomuţă I. Achim M. Vlase L. (2015). Development and optimization of quercetin-loaded PLGA nanoparticles by experimental design. Clujul Med. 88 (2), 214–223. 10.15386/cjmed-418 26528074 PMC4576773

[B53] Vandervoort J. Ludwig A. (2002). Biocompatible stabilizers in the preparation of PLGA nanoparticles: A factorial design study. Int. J. Pharm. 238 (1-2), 77–92. 10.1016/s0378-5173(02)00058-3 11996812

[B54] Vijayakumar M. R. Kosuru R. Vuddanda P. R. Singh S. K. Singh S. (2016). Trans resveratrol loaded DSPE PEG 2000 coated liposomes: An evidence for prolonged systemic circulation and passive brain targeting. J. drug Deliv. Sci. Technol. 33, 125–135. 10.1016/j.jddst.2016.02.009

[B55] Wanandi S. I. Limanto A. Yunita E. Syahrani R. A. Louisa M. Wibowo A. E. (2020). *In silico* and *in vitro* studies on the anticancer activity of andrographolide targeting survivin in human breast cancer stem cells. PloS one 15 (11), e0240020. 10.1371/journal.pone.0240020 33211707 PMC7676700

[B56] Wang R. Tang R. Li B. Ma X. Schnabl B. Tilg H. (2021). Gut microbiome, liver immunology, and liver diseases. Cell. Mol. Immunol. 18 (1), 4–17. 10.1038/s41423-020-00592-6 33318628 PMC7852541

[B57] Xiao-qiang W. Chu-sheng H. Hong-xing L. Yong-qiang H. E. (2009). Distribution, synthesis and biological activity of dalbergin. Nat. Prod. Res. Dev. 21 (5).

[B58] Zuco V. Supino R. Righetti S. C. Cleris L. Marchesi E. Gambacorti-Passerini C. (2002). Selective cytotoxicity of betulinic acid on tumor cell lines, but not on normal cells. Cancer Lett. 175 (1), 17–25. 10.1016/s0304-3835(01)00718-2 11734332

