# Supplementary figures and images for: Synthesis and appraisal of dalbergin-loaded PLGA nanoparticles modified with galactose against hepatocellular carcinoma: In-vitro, pharmacokinetic, and in-silico studies
Source: Front Pharmacol. 2022 Oct 28;13:1021867. doi: 10.3389/fphar.2022.1021867 (PMC9650263; doi:10.3389/fphar.2022.1021867)

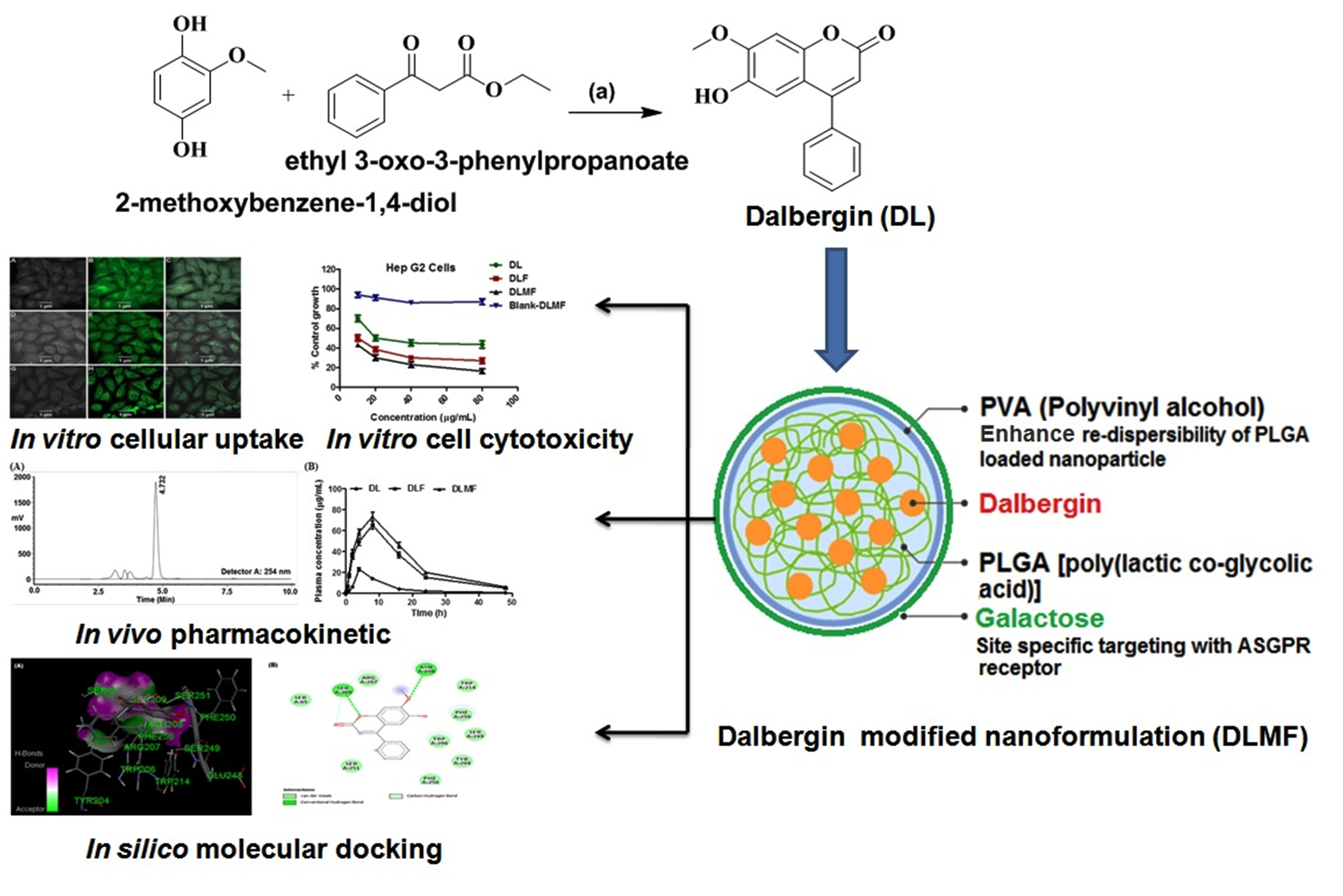

Supplement: Supplementary file 1 [file Image1.TIF]
