# Supplementary material for: Synthesis and appraisal of dalbergin-loaded PLGA nanoparticles modified with galactose against hepatocellular carcinoma: In-vitro, pharmacokinetic, and in-silico studies
Source: Front Pharmacol. 2022 Oct 28;13:1021867. doi: 10.3389/fphar.2022.1021867 (PMC9650263; doi:10.3389/fphar.2022.1021867)
Supplement: Supplementary file 2 [file DataSheet1.pdf]

Supplementary sheet

Synthesis and appraisal of dalbergin-loaded PLGA nanoparticles modified with galactose against hepatocellular carcinoma: *in-vitro*, pharmacokinetic, and *in-silico studies*

Anurag Kumar Gautam[1], Pranesh Kumar[1,2], Biswanath Maity[3], Ganesh Routholla[4], Balaram Ghosh[4], Kumarappan Chidambaram[5], M.Yasmin Begum[6], Adel Al Fatease[6], P.S. Rajinikanth[1], Sanjay Singh[1], Sudipta Saha[1†], Vijayakumar M.R[1*]

[1]Department of Pharmaceutical Sciences, Babasaheb Bhimrao Ambedkar University, Vidya Vihar, Lucknow, India.

[2]Department of Pharmacology, Aryakul College of Pharmacy & Research, Lucknow, India

[3]Centre of Biomedical Research, SGPGIMS Campus, Raebareli Road, Lucknow, India

[4]Department of Pharmacy, BITS-Pilani Hyderabad Campus, Hyderabad, India

[5]Department of Pharmacology and Toxicology, School of Pharmacy, King Khalid University, Abha, Saudi Arabia

[6]Department of Pharmaceutics, King Khalid University, Abha, Saudi Arabia

† In Memoriam of Dr. Sudipta Saha

[*]Author for Correspondence:

Dr. Vijayakumar M.R.

Assistant Professor

Department of Pharmaceutical Sciences,

Babasaheb Bhimrao Ambedkar University,

Vidya Vihar, Rai Bareli Road, Lucknow-226025, Uttar Pradesh, India.

Email: drvijayakumarmr@gmail.com

Figure A) ^{1}H-NMR SPECTROSCOPY

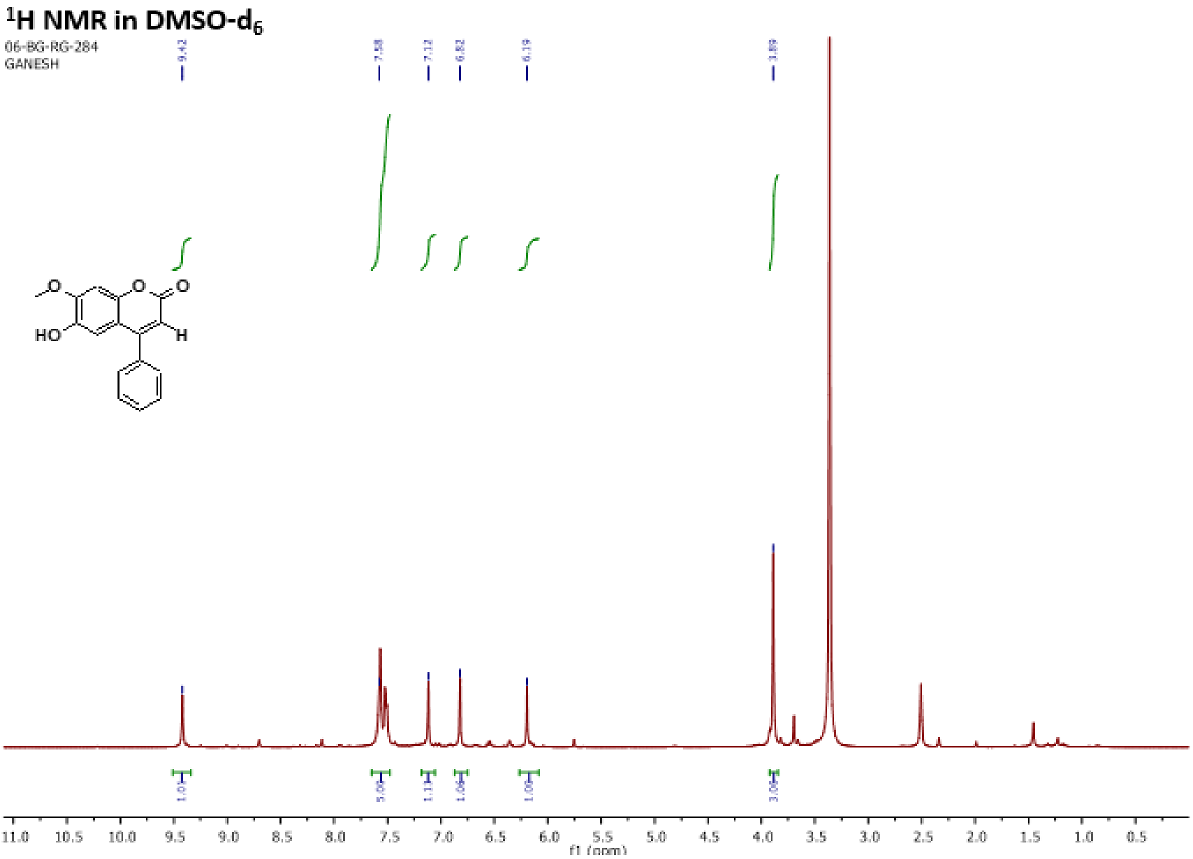

Figure B) ^{13}C-NMR SPECTROSCOPY

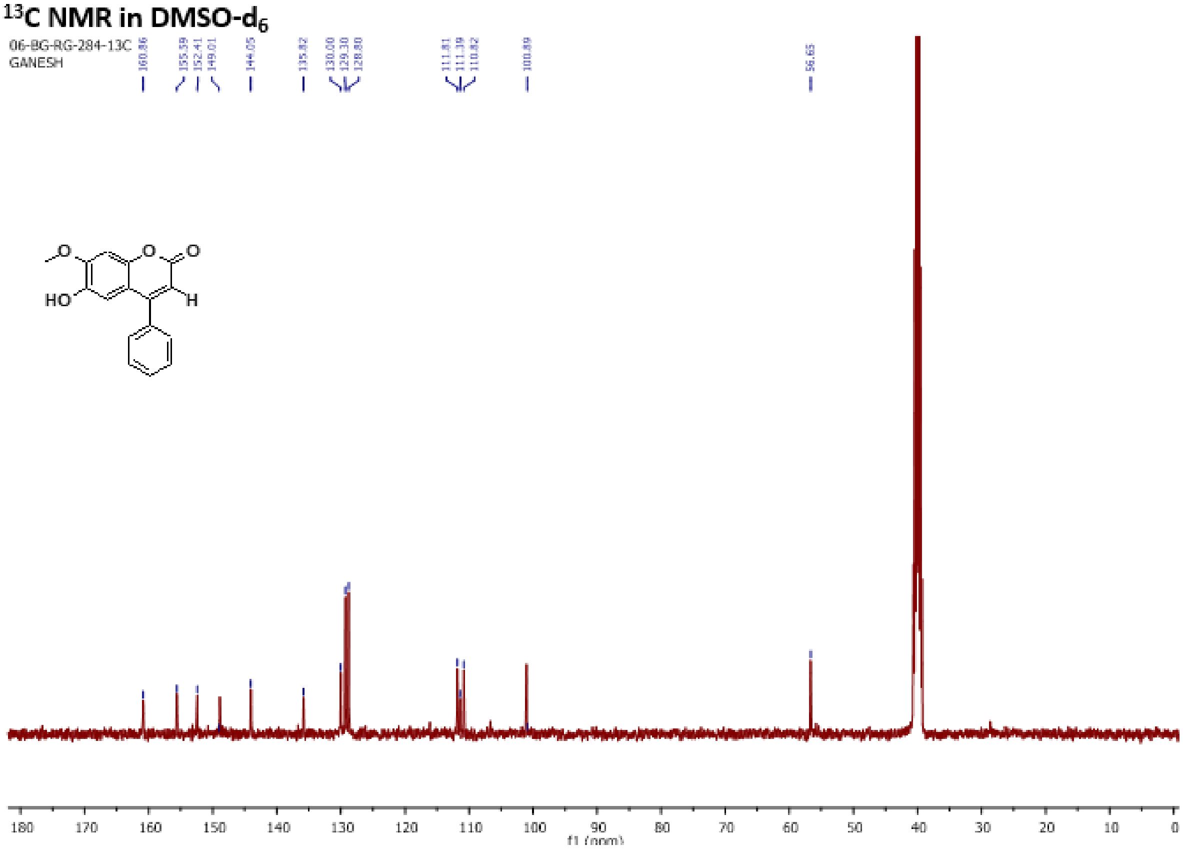

Figure C) LC-MASS SPECTROMETRY

Mass in methanol:

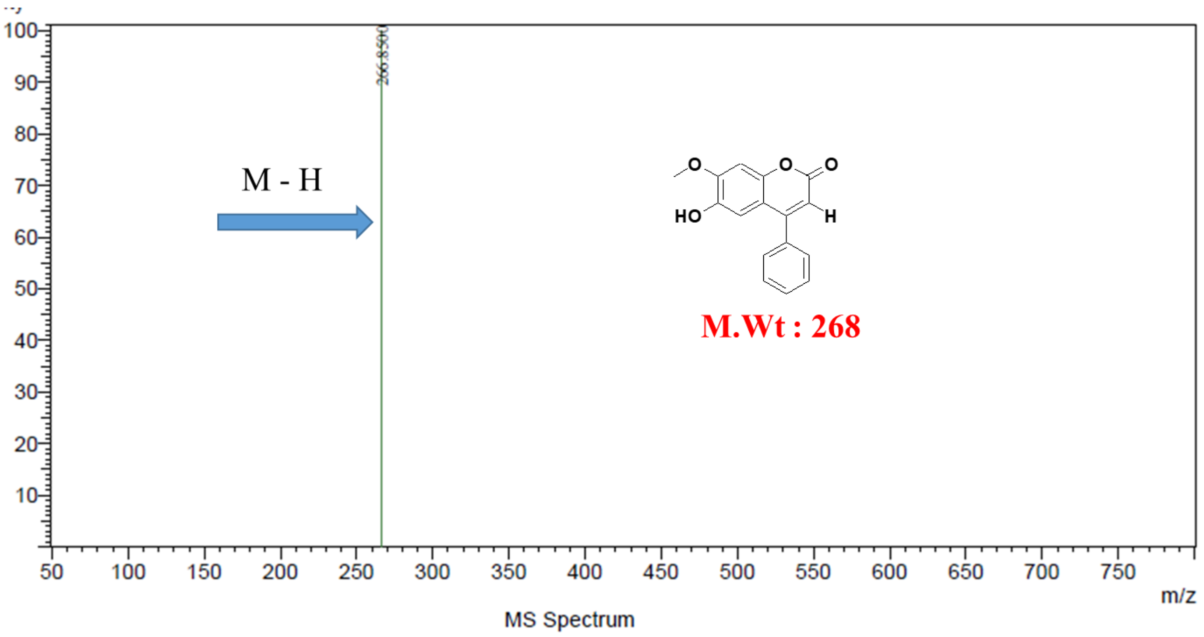

Table S1. Stability Studies of DLMF.

S.N.	Sampling interval (Days)	Particle Size (nm)		Entrapment Efficiency (%)	
		4°C±1°C	25°C±2°C	4°C±1°C	25°C±2°C
1	0th	118.04±2.45	122.12±2.23	93.14±2.02	96.56±2.76
2	30th	121.75±2.89	119.16±2.74	94.42±2.24	95.22±2.01
3	60th	123.12±2.29	123.23±2.26	92.23±2.12	94.43±1.98
4	90th	122.45±2.78	124.37±2.25	91.33±2.14	93.12±2.01
